# High-throughput characterization of genetic effects on DNA–protein binding and gene transcription

**DOI:** 10.1101/gr.237354.118

**Published:** 2018-11

**Authors:** Cynthia A. Kalita, Christopher D. Brown, Andrew Freiman, Jenna Isherwood, Xiaoquan Wen, Roger Pique-Regi, Francesca Luca

**Affiliations:** 1 Center for Molecular Medicine and Genetics, Wayne State University, Detroit, Michigan 48202, USA;; 2 Department of Genetics, University of Pennsylvania, Philadelphia, Pennsylvania 19104, USA;; 3 Department of Biostatistics, University of Michigan, Ann Arbor, Michigan 48109, USA;; 4 Department of Obstetrics and Gynecology, Wayne State University, Detroit, Michigan 48202, USA

## Abstract

Many variants associated with complex traits are in noncoding regions and contribute to phenotypes by disrupting regulatory sequences. To characterize these variants, we developed a streamlined protocol for a high-throughput reporter assay, Biallelic Targeted STARR-seq (BiT-STARR-seq), that identifies allele-specific expression (ASE) while accounting for PCR duplicates through unique molecular identifiers. We tested 75,501 oligos (43,500 SNPs) and identified 2720 SNPs with significant ASE (FDR < 10%). To validate disruption of binding as one of the mechanisms underlying ASE, we developed a new high-throughput allele-specific binding assay for NFKB1. We identified 2684 SNPs with allele-specific binding (ASB) (FDR < 10%); 256 of these SNPs also had ASE (OR = 1.97, *P*-value = 0.0006). Of variants associated with complex traits, 1531 resulted in ASE, and 1662 showed ASB. For example, we characterized that the Crohn's disease risk variant for rs3810936 increases NFKB1 binding and results in altered gene expression.

Genome-wide association studies (GWAS) have identified thousands of common genetic variants associated with complex traits, including normal traits and common diseases. Many GWAS hits are in noncoding regions, so the underlying mechanism leading to specific phenotypes is likely through disruption of gene regulatory sequence. Quantitative trait loci (QTLs) for molecular and cellular phenotypes (Dermitzakis 2012), such as gene expression (eQTL) (Brem and Kruglyak 2005; Stranger 2007; Innocenti et al. 2011; Wen et al. 2015; GTEx Consortium 2017), transcription factor binding (Kasowski et al. 2010), and DNase I sensitivity (dsQTL) (Degner et al. 2012) have been crucial in providing strong evidence and a better understanding of how genetic variants in regulatory sequences can affect gene expression levels (Melzer et al. 2008; Gibbs et al. 2010; Albert and Kruglyak 2015; GTEx Consortium 2017). In recent work, we were able to validate 48% of computationally predicted allelic effects on transcription factor binding through traditional reporter assays (Moyerbrailean et al. 2016b). However, traditional reporter assays are limited by the time and the cost of testing variants one at a time.

Massively parallel reporter assays (MPRA) have been developed for the simultaneous measurement of the regulatory function of thousands of constructs at once. For MPRA, a pool of synthesized DNA oligos containing a barcode at the 3′ UTR of a reporter plasmid is transfected into cells, and transcripts are isolated for RNA-seq. Expression driven by the synthesized enhancer region is estimated from the number of RNA reads normalized by the number of corresponding DNA reads (Kwasnieski et al. 2012, 2014; Melnikov et al. 2012; Patwardhan et al. 2012; Sharon et al. 2012). An alternative to MPRA is self-transcribing active regulatory region sequencing (STARR-seq) (Arnold et al. 2013), whose methods involve fragmenting the genome and cloning the fragments 3′ of the reporter gene. The approach is based on the concept that enhancers can function independently of their relative positions, so putative enhancers are placed downstream from a minimal promoter. Active enhancers transcribe themselves, with their strength quantified as the amount of RNA transcripts within the cell. Because they do not use separate barcodes, STARR-seq approaches have streamlined protocols that allow for higher throughput. Recently, high-throughput assays have been used to assess the enhancer function of genomic regions (Arnold et al. 2013; Wang et al. 2017), the allelic effects on gene expression for naturally occurring variation in 104 regulatory regions (Vockley et al. 2015), fine-map variants associated with gene expression in lymphoblastoid cell lines (LCLs) and HepG2 (Tewhey et al. 2016), and fine-map variants associated with red blood cell traits in GWAS (Ulirsch et al. 2016). These and other approaches with higher scalability and efficiency are necessary to validate and understand the validity of computational predictions and statistical associations for likely causal genetic variants.

In addition to using reporter assays to measure enhancer function on gene expression, there are several methods to directly measure binding affinity of DNA sequences for specific transcription factors. These methods include Spec-seq (Stormo et al. 2015), electrophoretic mobility shift assay-sequencing (EMSA-seq) (Wong et al. 2011), and Binding to Designed Library, Extracting, and sequencing (BUNDLE-seq) (Levo et al. 2015). In these assays, synthesized regions are combined in vitro with a purified transcription factor. The bound DNA-factor complexes are then isolated by polyacrylamide gel electrophoresis (PAGE), where sequencing of the derived libraries allows for quantification of the binding strength of regulatory regions. The benefit to these methods is that it is possible to assay any potential genetic variant of interest. In vivo methods (such as DNase-seq, ChIP-seq), instead, are limited to existing variation within a given sample. Also, in vivo methods cannot look at each transcription factor separately to identify the specific factor directly causing the change in binding, as the binding could be indirect with any number of cofactors. Although BUNDLE-seq compared binding and reporter gene expression, and EMSA has been previously used to ascertain allelic effects, none of the high-throughput EMSA methods have been used to determine allelic effects on binding.

## Results

**Figure 1.**
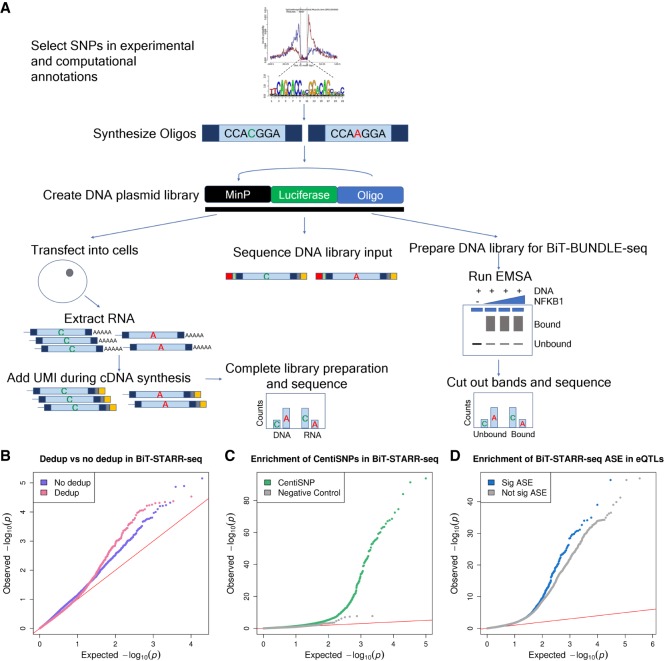
BiT-STARR-seq and BiT-BUNDLE-seq identify regulatory variants in noncoding regions. (*A*) Experimental outline. Oligos targeting the regulatory regions of interest (and either reference or alternate alleles) are designed to contain, on their ends, 15 bp matching the sequencing primers used for Illumina NGS. The DNA library is used both in the BiT-STARR-seq and BiT-BUNDLE-seq experiments. UMIs are added during cDNA synthesis for the BiT-STARR-seq RNA-seq library and prior to PAGE in the BiT-BUNDLE-seq protocol. (*B*) QQplot depicting the *P*-value distributions from QuASAR-MPRA for a single experimental replicate processed without removing duplicates (purple) or after removing duplicates using the UMIs (pink). (*C*) QQplot depicting the *P*-value distributions from the ASE test performed using QuASAR-MPRA on all replicates after removing duplicates. CentiSNPs are in green (Moyerbrailean et al. 2016b), and SNPs in the negative control group are in gray. (*D*) QQplot depicting the *P*-value distributions for eQTLs from Nédélec et al. (2016). SNPs with significant ASE (FDR < 10%) are in blue, and not significant ASE are in gray.

We have developed a new streamlined method called Biallelic Targeted STARR-seq (BiT-STARR-seq) to test for allele-specific effects in regulatory regions (Fig. 1A; Supplemental Fig. S1). We selected different categories of regulatory variants for this study, including eQTLs (Innocenti et al. 2011; Wen et al. 2015), CentiSNPs (Moyerbrailean et al. 2016b), ASB SNPs (Moyerbrailean et al. 2016b), variants associated with complex traits in GWAS (Pickrell 2014), and negative ASB controls (Moyerbrailean et al. 2016b) for a total of 50,609 SNPs (Methods). We designed two oligos targeting each of the alleles for a SNP and containing the regulatory region (200 bp) centered on the SNP (Fig. 1A; Supplemental Fig. S1; Methods). We also included the use of unique molecular identifiers (UMIs), added during cDNA synthesis. With these random UMIs we are in effect tagging identifiable replicates of the self-transcribing construct, which improves the analysis of the data by accounting for PCR duplicates. Our protocol also has the advantage of being highly streamlined. Unlike STARR-seq, our method does not require preparation of DNA regions for use in the assay, such as whole-genome fragmentation (Arnold et al. 2013) or targeting regions (Vanhille et al. 2015), although, similar to STARR-seq, it requires only a single cloning and transformation step. Because the UMIs are inserted after transfection, there are no additional bottleneck issues (due to library complexity) in the cloning and transformation steps.

We generated seven replicates of the DNA library, which were highly and significantly correlated (Spearman's *ρ* = [0.97, 0.98], *P*-value <0.01) (Supplemental Fig. S2). The DNA library was then transfected in LCLs (nine biological replicates), and we were able to recover a total of 43,500 testable SNPs (for RNA counts filter, see Methods). Read counts for the nine biological replicates were highly correlated (Spearman's *ρ* = [0.35, 0.72], *P*-value <0.01) (Supplemental Fig. S3). To identify SNPs with allele-specific effects, we applied QuASAR-MPRA (Kalita et al. 2018). For each SNP, the reference and alternate allele counts were compared to the DNA proportion in the plasmid library. We identified a total of 2720 SNPs with ASE from the combined replicates (FDR < 10%) (Supplemental Table S1). To investigate the importance of UMIs in this experimental approach, we reanalyzed our data without removing duplicates. For the combined replicates, inflation (genomic inflation test) (Methods) is greatly increased (from 1.10 to 1.73). If only a single RNA library replicate is considered, the number of detected ASE is about fourfold reduced (Fig. 1B).

SNPs with ASE are significantly enriched for variants predicted to impact transcription factor binding (CentiSNPs) (Fisher's exact test OR = 2.49, *P*-value = 4.55 × 10^−6^) (Fig. 1C; Supplemental Fig. S4; Supplemental Tables S2, S5; Moyerbrailean et al. 2016b). Additionally, SNPs with ASE are enriched for low *P*-values in an eQTL mapping study performed in immune cells (Fig. 1D; Supplemental Table S4; Nédélec et al. 2016), thus confirming that our synthetic oligos can reproduce allele-specific regulatory effects observed in a native (non-episomal) cellular context.

### Motif, regulatory region size, and chromatin context effects

**Figure 2.**
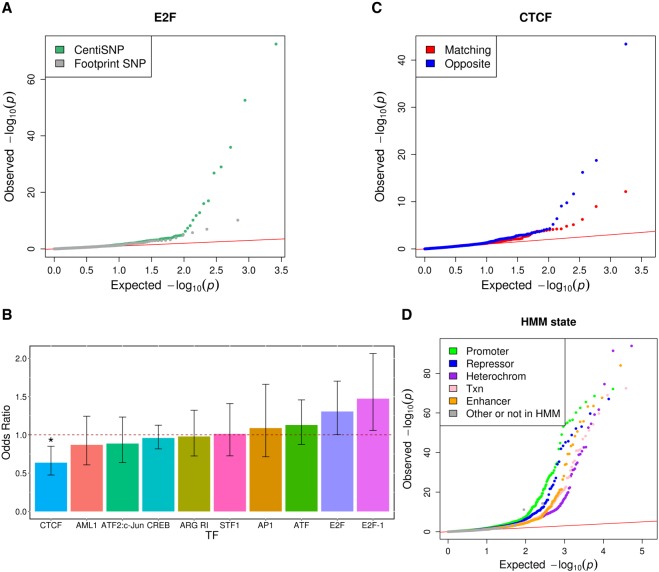
ASE for individual transcription factors. (*A*) QQplot depicting the ASE *P*-value distributions from QuASAR-MPRA, for SNPs overlapping with E2F footprint annotations. SNPs predicted to alter binding (CentiSNPs) are represented in green, and SNPs that are in E2F but predicted to have no effect on binding are in gray. (*B*) Enrichment for ASE in individual transcription factor binding sites calculated when motif strand matched the BiT-STARR-seq oligo transcription direction. Odds ratio (*y*-axis) for each transcription factor tested (*x*-axis) is shown in the bar plot; error bars are the 95% CI from the Fisher's exact test. Odds ratios *below* the dotted line represent enrichment for opposite direction oligo/motif configuration. Stars are shown *above* significant results (Bonferroni adjusted *P*-value *<*0.05). (*C*) QQplot depicting the ASE *P*-value distributions from QuASAR-MPRA, overlapping with CTCF footprint annotations. The SNPs where the motif strand matches the BiT-STARR-seq oligo direction relative to the TSS are in red, and blue shows where the motif strand is the opposite of the BiT-STARR-seq direction. (*D*) QQplot depicting the ASE *P*-value distributions from QuASAR-MPRA, overlapping with chromatin state annotations.

The CentiSNP annotation is informative of the specific transcription factor motif being disrupted by a SNP. By leveraging this information, we were able to analyze allelic effects for specific transcription factor motifs (Fig. 2A; Supplemental Table S6). Additionally, by combining the ASE results with the direction of the motif, we can characterize whether the motif is active in both directions or only in one direction. This would suggest that some TF binding motifs tend to function specifically in one direction. We found that when both alleles are covered in both directions, the allelic effects on gene expression tend to agree in direction and magnitude. If we categorize these directional allelic effects per motif, we do not observe major differences with the notable exception of CTCF (Fig. 2B). Specifically, we find that SNPs in footprints for CTCF are significantly enriched (Fisher's exact test OR = 1.57, Bonferroni *P* = 0.02) for ASE when the direction of transcription of the reporter gene is opposite to that of the motif strand (Fig. 2C).

Although oligos were designed to have the variant centered in the middle of the synthesized region, this does not necessarily mean that the SNP is centered in a DNase window (Supplemental Fig. S1). Although position within the window does not affect the ASE signal, the main effect seems to depend on the presence/absence of the tested site within the DNase window (Supplemental Fig. S5) and, in a lesser degree, it depends on the peak size (Supplemental Fig. S6). SNPs were originally selected based on the CentiSNP annotation, but when we considered chromatin states (Broad ChromHMM state), we detect enrichment for promoter state among SNPs with ASE (Fig. 2D).

### Allele-specific binding for NFKB1

To understand the effect of a regulatory variant on complex traits, it is necessary to first dissect the molecular function that is impacted by the nucleotide change. The CentiSNP prediction provides specific hypotheses for allelic effects on transcription factor binding that can be directly tested through experimentation. Further matching ASB to ASE identified in BiT-STARR-seq would provide a complete molecular mechanism, from predicted binding effects, to experimentally validated binding effects, to validated effects on expression. Because of the enrichment of CentiSNPs among SNPs with ASE in BiT-STARR-seq, we performed BiT-BUNDLE-seq to validate their effect on transcription factor binding. This is a new and efficient extension of high-throughput reporter assays, because it uses the same input DNA library. We performed BiT-BUNDLE-seq with purified NFKB1 (at three different concentrations), which is an important regulator of the immune response in LCLs and other immune cells (Li and Verma 2002; Beinke and Ley 2004; Smale 2010). Previous studies have successfully identified ASB from ChIP-seq for all NF-κB subunits in LCLs (Martone et al. 2003; Lim et al. 2007; Heinz et al. 2010; Kasowski et al. 2010; Zhao et al. 2014; Jin et al. 2013) and NFKB1 footprints are induced in response to infection (Pacis et al. 2015). Additionally, NF-κB complex was found to be 50-fold enriched for reQTLs from response to *Listeria* and *Salmonella* (Nédélec et al. 2016).

**Figure 3.**
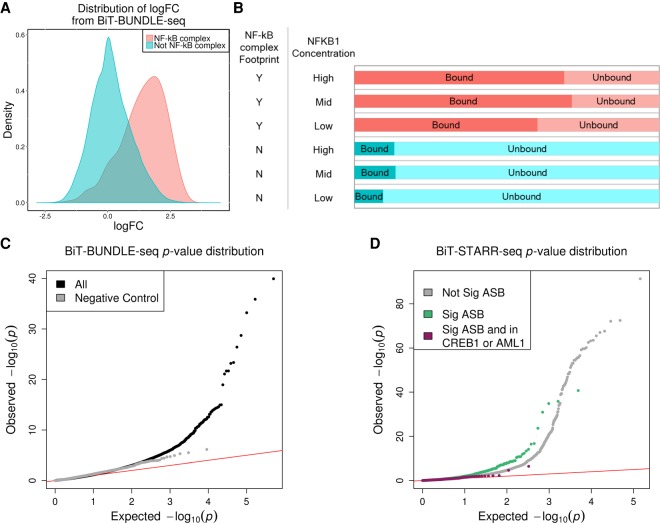
Allele-specific binding for NFKB1. (*A*) Density plot of the logFC (from DESeq2) between bound and unbound DNA fractions from the BiT-BUNDLE-seq experiment. Regions in red are those containing a SNP in a NF-κB complex footprint; regions in blue are those containing a SNP in footprints for other transcription factors. (*B*) Bar plot representing the number of independent enhancer regions in bound (dark color, DESeq2 logFC > 1 and FDR < 1%) and unbound (light color) DNA. NFKB1 concentration and presence of a NF-κB complex footprint are indicated in the two columns on the *left* of the panel. (*C*) QQplot depicting the *P*-value distributions from testing for ASB signal specific to the bound DNA fraction using ΔAST (black) and SNPs in the negative control group (gray). (*D*) QQplot depicting the ASE *p*-value distribution from QuASAR-MPRA for SNPs with significant (FDR < 10%) ASB (green), SNPs with significant (FDR < 10%) ASB and are also in CREB1 or AML1 footprints (maroon), or not significant ASB (gray) in the BiT-BUNDLE-seq experiment.

We first analyzed NFKB1 binding between the bound and unbound libraries and identified 9361 significantly (logFC > 1 and FDR < 1%) overrepresented regions in the bound library (Supplemental Tables S7–S10). As expected, these regions were enriched (OR = 11.70–13.75, *P*-value = 7.95 × 10^−27^ to 1.23 × 10^−15^) for NF-κB complex footprints (Fig. 3A; Supplemental Fig. S7), with a higher portion of these regions in the middle concentration of NFKB1 compared with the low or high concentrations (Fig. 3B). We hypothesize that this is because the low concentration does not capture all of the NFKB1 binding, whereas the high concentration likely results in nonspecific binding. We then used ΔAST (Moyerbrailean et al. 2016a) to identify ASB in the bound library (compared with the unbound library) and combined the replicates using Stouffer's method (Methods; Fig. 3C). We successfully identified 386 SNPs at low concentration, 797 SNPs at middle concentration, and 894 SNPs at high concentration with significant ASB at FDR < 10% (Fig. 3D; Supplemental Fig. S8), for a total of 2684 SNPs when aggregating all experiments (Supplemental Tables S3, S11). These spanned our designed regulatory categories, with the greatest number covering CentiSNPs (Supplemental Table S12). When we considered these ASB SNPs in combination with the ASE results from the BiT-STARR-seq (Supplemental Fig. S9), we found that SNPs with ASE are enriched for NFKB1 ASB (Fisher's exact test, OR = 2.04, *P*-value = 1.51 × 10^−16^) (Fig. 3D). For ASB variants not showing ASE, we found that there is enrichment for these being in either the CREB1 or AML1 motifs (Fig. 3D; Supplemental Fig. S10; for Fisher's test, see Supplemental Table S13), which are factors known to antagonize NF-κB complex binding (Ollivier et al. 1996; Parry and Mackman 1997; Nakagawa et al. 2009, 2011).This confirms our hypothesis that disruption of NFKB1 binding is one of the mechanisms underlying allele-specific expression in our data set.

### Overlap with signals from GWAS

**Figure 4.**
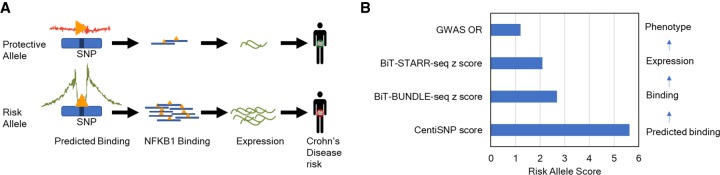
Overlap with GWAS. (*A*) Integration of prediction, BiT-BUNDLE-seq, BiT-STARR-seq, and GWAS results for Crohn's disease risk variant rs3810936. Triangles represent transcription factors. (*B*) A comparison of allelic effects from computational prediction to phenotype for rs3810936. Predicted log odds score is the reference prior log odds–alternate log odds from the CentiSNP annotation. BiT-BUNDLE-seq *Z*-score is the *Z*-score from the metaanalysis of ASB from all three concentrations of NFKB1. BiT-STARR-seq *Z*-score is the *Z*-score from metaanalysis of ASE for nine experimental replicates. GWAS OR is the odds ratio from rs3810936 alternate allele with Crohn's disease (Yamazaki et al. 2005; Franke et al. 2010; Baskaran et al. 2014; Lee et al. 2015). All scores are signed relative to the risk allele, which is the alternate allele.

We used ASB and ASE in combination with transcription factor binding motifs to assign mechanistic function to putatively causal SNPs linked to complex traits. We found 2054 CentiSNPs with ASB (*P*-value <0.05) and 1769 CentiSNPs with ASE (*P*-value <0.05) associated to a complex trait in the GWAS catalog (Supplemental Tables S14, S15) or from fgwas (Moyerbrailean et al. 2016b). Considering all SNPs tested, there are 173 SNPs that have both ASB and ASE (FDR < 10%), and 164 of them (95%) are also associated with a complex trait. We were able to dissect the molecular mechanism for rs3810936, a variant associated with risk for Crohn's disease in multiple populations (Fig. 4A,B; Yamazaki et al. 2005; Franke et al. 2010; Baskaran et al. 2014; Lee et al. 2015). This variant is a CentiSNP for the factor HMX3 (also known as NKX5-1), and we find ASB for NFKB1 (*P*-value = 0.006) in the BiT-BUNDLE-seq assay and ASE (*P*-value = 0.034) in both directions in the BiT-STARR-seq. This SNP is a synonymous variant in gene *TNFSF15* (also known as *TL1A*), which encodes for a cytokine that belongs to the tumor necrosis factor (TNF) ligand family.

Increased TL1A expression has been reported in inflamed Crohn's disease tissue, compared with noninflamed areas, and in ulcerative colitis patient serum (Bamias et al. 2003, 2010; Prehn et al. 2004). TL1A gives costimulatory signals to activated lymphocytes through binding to TNFRSF25 (also known as DR3) (Migone et al. 2002), which induces the secretion of interferon gamma (IFNG) (Prehn et al. 2004; Papadakis et al. 2005). This gene modulates Th-1 and Th-17 (Bamias et al. 2003; Takedatsu et al. 2008), creating an immunological state that leads to the mucosal inflammation of Crohn's disease. Stimulation of the TL1A pathway, in monocytes and T cells from patients carrying the disease-associated *TL1A* SNPs, showed higher levels of TL1A expression, therefore aberrant *TL1A* expression may be a factor driving IBD development (Kakuta et al. 2009; Michelsen et al. 2009). In addition, this gene has been found to be down-regulated in response to dexamethasone (Moyerbrailean et al. 2016a), a corticosteroid used to treat many inflammatory and autoimmune conditions. Although this variant is not found in ChIP-seq from ENCODE, ENCODE studies used RELA (also known as p65) for NF-κB subunit, where our study used NFKB1 (also known as p50). We therefore identify a novel variant that disrupts binding of NFKB1, where the alternate allele (C) has increased binding. This leads to an increase in gene expression for the alternate allele, which is also the risk allele for Crohn's disease (OR = 1.21, *P*-value = 1 × 10^−15^).

## Discussion

The recent adaptation of MPRA to investigate ASE allows for validation of regulatory variants in transcription factor binding sites, which have been shown to be functionally relevant to fine-map eQTLs (Tewhey et al. 2016) and GWAS signals (Ulirsch et al. 2016). However, the use of functional genomics to select relevant regions prior to experimental validation can reduce the number of sites it is necessary to validate. We developed a high-throughput reporter assay that synthesizes these selected regions (similar to MPRA), clones them in 3′ of the reporter gene (similar to STARR-seq), and includes the addition of a UMI during cDNA synthesis (new to our protocol). This is the most streamlined protocol to date and allows for removal of PCR duplicates, which reduces noise in the data for greater power to detect ASE.

Our results show that using existing annotations to prioritize regulatory variants for high-throughput reporter assays is an effective strategy. The CentiSNP annotation, in particular, contains information that can be used to analyze ASB/ASE for individual transcription factor motifs and investigate potential molecular mechanisms of action. We found that direction is an important factor in the case of CTCF, most likely due to how CTCF functions as an insulator between the enhancer and the promoter when they are in anti-parallel directions. Previous studies have shown that CTCF, a well-characterized insulator, has binding sites at the anchors of chromatin loops. These are arranged in forward–reverse orientations (Guo et al. 2012, 2015; Monahan et al. 2012; Alt et al. 2013; Rao et al. 2014; Vietri Rudan et al. 2015), where the relative positions and orientations of the binding sites are important for the mechanism of action (Guo et al. 2015). In our case, the interaction could be mediated either by the basal transcriptional machinery at the TSS and/or an additional weak CTCF binding site (M01259) that is present in the promoter and could help to establish a DNA loop. However, there may be alternative explanations for this result because reporter assays may not reflect the native regulatory landscape in human cells (Huerfano et al. 2013; Muerdter et al. 2017).

Generally, caution should be used in interpreting reporter assay gene expression differences across cell types, because transfection may perturb the cell state. However, it is important to highlight that any *trans*-acting effects (e.g., promoter strength, type 1 interferon response activation) should affect both alleles similarly and therefore should not induce false positives in the allele-specific signal.

We used our library of oligos also in a BiT-BUNDLE-seq assay for identification of ASB for NFKB1. This is a novel approach to combine ASB and ASE identification in high-throughput assays using the same sequences. Our results show that this integration is a useful approach to validate the molecular mechanism for specific transcription factors. Allelic effects on transcription factor binding and gene expression are not always concordant. Some of this discordance is due to lack of power to detect ASB/ASE overlap, as well as other technical considerations. For example, in BiT-BUNDLE-seq, only one single TF (NFKB1) is available for binding, whereas in BiT-STARR-seq, other cofactors are present in the cell to affect binding. Additionally, there can be discordance in direction of effect, where, for example, an allele can lead to increased binding of a factor with repressing activity on gene expression (e.g., variants in CREB1/AML1 binding sites). These regulatory events are likely to be captured in the BiT-STARR-seq assay, which is performed in LCLs where CREB1, AML1, and NFKB1 are active. These results highlight that multiple types of assays are necessary to capture the detailed molecular mechanism of gene regulation. Additionally, integration with GWAS can identify and further characterize the molecular mechanisms linking causal genetic variants with complex traits.

## Methods

### BiT-STARR-seq

Supplemental Tables S2–S4 report the annotations we have considered with their sources, and Supplemental Tables S16 and S12 include the library composition. Each regulatory region was designed to have two oligos: one for each of the alleles. DNA inserts 230 bp long, corresponding to 200 bp of regulatory sequence, were synthesized by Agilent to contain the regulatory region and the SNP of interest within the first 150 bp (Supplemental Fig. S1). We performed a first round of PCR to generate double-stranded oligos and complete the sequencing primers, followed by a subsequent round of PCR to amplify the material. Plasmid pGL4.23 (Promega) was linearized using CloneAmp HiFi PCR Premix (Clontech) primers [STARR_F_SH and STARR_R_SH]. Inserts were cloned into the linear plasmid using standard In-Fusion (Clontech) cloning protocol. Clones (Supplemental Methods, BiT-STARR-seq plasmid) were transformed into XL10-Gold Ultracompetent Cells (Agilent) in a total of seven reactions. DNA was extracted using EndoFree maxiprep kit (Qiagen).

The DNA library was transfected into LCLs (GM18507) using standard nucleofection protocol, program DS150. We performed nine biological replicates of the transfection from seven independent cell cultures. After transfection, cells were incubated at 37°C and 5% CO_2_ in RPMI1640 with 15% FBS and 1% Gentamycin for 24 h. Cell pellets were then lysed using RLT lysis buffer (Qiagen) and cryopreserved at −80°C. For RNA libraries, total RNA was isolated from the thawed lysates using RNeasy Plus Mini Kit (Qiagen). RNA-seq libraries from the polyadenylated RNA were prepared using a custom protocol described in the Supplemental Methods (library preparation). We prepared seven replicates of the DNA library using a modified version of the PCR protocol as previously described (Buenrostro et al. 2013; Supplemental Methods, library preparation).

### BiT-BUNDLE-seq

We developed BiT-BUNDLE-seq, by modifying the design of the BUNDLE-seq protocol (Levo et al. 2015). Specifically, input DNA sequences were extracted from the BiT-STARR-seq DNA plasmid library. We used N-terminal GST-tagged, recombinant human NFKB1 from EMD Millipore. Experiments were performed in triplicates for each NFKB1 concentration. Libraries extracted from the bound and unbound DNA bands after PAGE were quantified and loaded on the Illumina NextSeq 500 for sequencing. The full-length protocol can be found in Supplemental Methods (BiT-STARR-seq protocol).

### Data processing

Reads were mapped using the HISAT2 aligner (Kim et al. 2015), using the “genome_snp” GRCh37 index (ftp://ftp.ccb.jhu.edu/pub/infphilo/hisat2/data/grch37_snp.tar.gz) so as to avoid reference bias. Realigning the reads to GRCh38 should not affect the conclusions as any problematic region of the genome is excluded from any analysis (Supplemental Methods, oligo selection and design). We then ran UMItools (Smith et al. 2017) using standard flags to remove duplicates. To identify SNPs with allele-specific effects, we applied QuASAR-MPRA (Kalita et al. 2018), in which for each SNP the reference and alternate allele counts were compared to the DNA proportion. QuASAR-MPRA results from each replicate were then combined using the fixed effects method and corrected for multiple tests (Benjamini and Hochberg 1995).

Each replicate for the bound and unbound libraries from BiT-BUNDLE-seq were run through QuASAR-MPRA using the calculated reference proportion (combined unbound and bound DNA). These were then compared using ΔAST (Moyerbrailean et al. 2016a) to identify ASB in the bound fraction that is differential relative to the unbound fraction. The replicates were combined using Stouffer's method (Stouffer et al. 1949) to identify ASB for each NFKB1 concentration and combined again to identify the total ASB. Libraries were additionally analyzed with DESeq2 (Love et al. 2014) to discriminate between bound and unbound constructs.

## Data access

The sequencing data from this study have been submitted to the NCBI Sequence Read Archive (SRA; https://www.ncbi.nlm.nih.gov/sra) under accession number SRP154945.

## Supplementary Material

Supplemental Material

## References

[GR237354KALC1] Albert FW, Kruglyak L. 2015 The role of regulatory variation in complex traits and disease. Nat Rev Genet 16: 197–212. 25707927 10.1038/nrg3891

[GR237354KALC2] Alt F, Zhang Y, Meng FL, Guo C, Schwer B. 2013 Mechanisms of programmed DNA lesions and genomic instability in the immune system. Cell 152: 417–429. 23374339 10.1016/j.cell.2013.01.007 PMC4382911

[GR237354KALC3] Arnold C, Gerlach D, Stelzer C, Boryń Ł. 2013 Genome-wide quantitative enhancer activity maps identified by STARR-seq. Science 339: 1074–1077. 23328393 10.1126/science.1232542

[GR237354KALC4] Bamias G, Martin C, Marini M, Hoang S, Mishina M, Ross WG, Sachedina MA, Friel CM, Mize J, Bickston SJ, 2003 Expression, localization, and functional activity of TL1A, a novel Th1-polarizing cytokine in inflammatory bowel disease. J Immunol 171: 4868–4874. 14568967 10.4049/jimmunol.171.9.4868

[GR237354KALC5] Bamias G, Kaltsa G, Siakavellas SI, Papaxoinis K, Zampeli E, Michopoulos S, Zouboulis-Vafiadis I, Ladas SD. 2010 High intestinal and systemic levels of decoy receptor 3 (DcR3) and its ligand TL1A in active ulcerative colitis. Clin Immunol 137: 242–249. 20675196 10.1016/j.clim.2010.07.001

[GR237354KALC6] Baskaran K, Pugazhendhi S, Ramakrishna BS. 2014 Protective association of tumor necrosis factor superfamily 15 (TNFSF15) polymorphic haplotype with ulcerative colitis and Crohn's disease in an Indian population. PLoS One 9: e114665. 25501099 10.1371/journal.pone.0114665 PMC4264777

[GR237354KALC7] Beinke S, Ley SC. 2004 Functions of NF-κB1 and NF-κB2 in immune cell biology. Biochem J 382: 393–409. 15214841 10.1042/BJ20040544 PMC1133795

[GR237354KALC8] Benjamini Y, Hochberg Y. 1995 Controlling the false discovery rate: a practical and powerful approach to multiple testing. J R Stat Soc Series B 57: 289–300.

[GR237354KALC9] Brem RB, Kruglyak L. 2005 The landscape of genetic complexity across 5,700 gene expression traits in yeast. Proc Natl Acad Sci 102: 1572–1577. 15659551 10.1073/pnas.0408709102 PMC547855

[GR237354KALC10] Buenrostro J, Giresi P, Zaba L. 2013 Transposition of native chromatin for fast and sensitive epigenomic profiling of open chromatin, DNA-binding proteins and nucleosome position. Nat Methods 10: 1213–1218. 24097267 10.1038/nmeth.2688 PMC3959825

[GR237354KALC11] Degner JF, Pai AA, Pique-Regi R, Veyrieras JB, Gaffney DJ, Pickrell JK, De Leon S, Michelini K, Lewellen N, Crawford GE, 2012 DNaseI sensitivity QTLs are a major determinant of human expression variation. Nature 482: 390–394. 22307276 10.1038/nature10808 PMC3501342

[GR237354KALC12] Dermitzakis E. 2012 Cellular genomics for complex traits. Nat Rev Genet 13: 215–220. 22330769 10.1038/nrg3115

[GR237354KALC13] Franke A, McGovern DP, Barrett JC, Wang K, Radford-Smith GL, Ahmad T, Lees CW, Balschun T, Lee J, Roberts R, 2010 Genome-wide meta-analysis increases to 71 the number of confirmed Crohn's disease susceptibility loci. Nat Genet 42: 1118–1125. 21102463 10.1038/ng.717 PMC3299551

[GR237354KALC14] Gibbs J, van der Brug M, Hernandez D. 2010 Abundant quantitative trait loci exist for DNA methylation and gene expression in human brain. PLoS Genet 6: e1000952. 20485568 10.1371/journal.pgen.1000952 PMC2869317

[GR237354KALC15] GTEx Consortium. 2017 Genetic effects on gene expression across human tissues. Nature 550: 204–213. 29022597 10.1038/nature24277 PMC5776756

[GR237354KALC16] Guo Y, Monahan K, Wu H, Gertz J, Varley KE, Li W, Myers RM, Maniatis T, Wu Q. 2012 CTCF/cohesin-mediated DNA looping is required for protocadherin *α* promoter choice. Proc Natl Acad Sci 109: 21081–21086. 23204437 10.1073/pnas.1219280110 PMC3529044

[GR237354KALC17] Guo Y, Xu Q, Canzio D, Shou J, Li J, Gorkin D, Jung I, Wu H, Zhai Y, Tang Y, 2015 CRISPR inversion of CTCF sites alters genome topology and enhancer/promoter function. Cell 162: 900–910. 26276636 10.1016/j.cell.2015.07.038 PMC4642453

[GR237354KALC18] Heinz S, Benner C, Spann N, Bertolino E, Lin YC, Laslo P, Cheng JX, Murre C, Singh H, Glass CK. 2010 Simple combinations of lineage-determining transcription factors prime *cis*-regulatory elements required for macrophage and B cell identities. Mol Cell 38: 576–589. 20513432 10.1016/j.molcel.2010.05.004 PMC2898526

[GR237354KALC19] Huerfano S, Ryabchenko B, Forstová J. 2013 Nucleofection of expression vectors induces a robust interferon response and inhibition of cell proliferation. DNA Cell Biol 32: 467–479. 23745681 10.1089/dna.2012.1950 PMC3725941

[GR237354KALC20] Innocenti F, Cooper G, Stanaway I. 2011 Identification, replication, and functional fine-mapping of expression quantitative trait loci in primary human liver tissue. PLoS Genet 7: e1002078. 21637794 10.1371/journal.pgen.1002078 PMC3102751

[GR237354KALC21] Jin F, Li Y, Dixon JR, Selvaraj S, Ye Z, Lee AY, Yen CA, Schmitt AD, Espinoza CA, Ren B. 2013 A high-resolution map of the three-dimensional chromatin interactome in human cells. Nature 503: 290–294. 24141950 10.1038/nature12644 PMC3838900

[GR237354KALC22] Kakuta Y, Ueki N, Kinouchi Y, Negoro K, Endo K, Nomura E, Takagi S, Takahashi S, Shimosegawa T. 2009 *TNFSF15* transcripts from risk haplotype for Crohn's disease are overexpressed in stimulated T cells. Hum Mol Genet 18: 1089–1098. 19124533 10.1093/hmg/ddp005

[GR237354KALC23] Kalita CA, Moyerbrailean GA, Brown C, Wen X, Luca F, Pique-Regi R. 2018 QuASAR-MPRA: accurate allele-specific analysis for massively parallel reporter assays. Bioinformatics 34: 787–794. 29028988 10.1093/bioinformatics/btx598 PMC6049023

[GR237354KALC24] Kasowski M, Grubert F, Heffelfinger C. 2010 Variation in transcription factor binding among humans. Science 328: 232–235. 20299548 10.1126/science.1183621 PMC2938768

[GR237354KALC25] Kim D, Langmead B, Salzberg SL. 2015 HISAT: a fast spliced aligner with low memory requirements. Nat Methods 12: 357–360. 25751142 10.1038/nmeth.3317 PMC4655817

[GR237354KALC26] Kwasnieski J, Mogno I, Myers C. 2012 Complex effects of nucleotide variants in a mammalian *cis*-regulatory element. Proc Natl Acad Sci 109: 19498–19503. 23129659 10.1073/pnas.1210678109 PMC3511131

[GR237354KALC27] Kwasnieski J, Fiore C, Chaudhari H, Cohen B. 2014 High-throughput functional testing of ENCODE segmentation predictions. Genome Res 24: 1595–1602. 25035418 10.1101/gr.173518.114 PMC4199366

[GR237354KALC28] Lee YJ, Kim KM, Jang JY, Song K. 2015 Association of *TNFSF15* polymorphisms in Korean children with Crohn's disease. Pediatr Int 57: 1149–1153. 25998826 10.1111/ped.12686

[GR237354KALC29] Levo M, Zalckvar E, Sharon E, Dantas Machado AC, Kalma Y, Lotam-Pompan M, Weinberger A, Yakhini Z, Rohs R, Segal E. 2015 Unraveling determinants of transcription factor binding outside the core binding site. Genome Res 25: 1018–1029. 25762553 10.1101/gr.185033.114 PMC4484385

[GR237354KALC30] Li Q, Verma IM. 2002 NF-κB regulation in the immune system. Nat Rev Immunol 2: 725–734. 12360211 10.1038/nri910

[GR237354KALC31] Lim CA, Yao F, Wong JJY, George J, Xu H, Chiu KP, Sung WK, Lipovich L, Vega VB, Chen J, 2007 Genome-wide mapping of RELA(p65) binding identifies E2F1 as a transcriptional activator recruited by NF-κB upon TLR4 activation. Mol Cell 27: 622–635. 17707233 10.1016/j.molcel.2007.06.038

[GR237354KALC32] Love MI, Huber W, Anders S. 2014 Moderated estimation of fold change and dispersion for RNA-seq data with DESeq2. Genome Biol 15: 550. 25516281 10.1186/s13059-014-0550-8 PMC4302049

[GR237354KALC33] Martone R, Euskirchen G, Bertone P, Hartman S, Royce TE, Luscombe NM, Rinn JL, Nelson FK, Miller P, Gerstein M, 2003 Distribution of NF-*κ*B-binding sites across human chromosome 22. Proc Natl Acad Sci 100: 12247–12252. 14527995 10.1073/pnas.2135255100 PMC218744

[GR237354KALC34] Melnikov A, Murugan A, Zhang X, Tesileanu T. 2012 Systematic dissection and optimization of inducible enhancers in human cells using a massively parallel reporter assay. Nat Biotechnol 30: 271–277. 22371084 10.1038/nbt.2137 PMC3297981

[GR237354KALC35] Melzer D, Perry J, Hernandez D, Corsi A. 2008 A genome-wide association study identifies protein quantitative trait loci (pQTLs). PLoS Genet 4: e1000072. 18464913 10.1371/journal.pgen.1000072 PMC2362067

[GR237354KALC36] Michelsen KS, Thomas LS, Taylor KD, Yu QT, Mei L, Landers CJ, Derkowski C, McGovern DPB, Rotter JI, Targan SR. 2009 IBD-associated *TL1A* gene (*TNFSF15*) haplotypes determine increased expression of TL1A protein. PLoS One 4: e4719. 19262684 10.1371/journal.pone.0004719 PMC2648040

[GR237354KALC37] Migone TS, Zhang J, Luo X, Zhuang L, Chen C, Hu B, Hong JS, Perry JW, Chen SF, Zhou JX, 2002 TL1A is a TNF-like ligand for DR3 and TR6/DcR3 and functions as a T cell costimulator. Immunity 16: 479–492. 11911831 10.1016/s1074-7613(02)00283-2

[GR237354KALC38] Monahan K, Rudnick ND, Kehayova PD, Pauli F, Newberry KM, Myers RM, Maniatis T. 2012 Role of CCCTC binding factor (CTCF) and cohesin in the generation of single-cell diversity of protocadherin-*α* gene expression. Proc Natl Acad Sci 109: 9125–9130. 22550178 10.1073/pnas.1205074109 PMC3384188

[GR237354KALC39] Moyerbrailean G, Richards A, Kurtz D, Kalita CA, Davis G, Harvey C, Alazizi A, Watza D, Sorokin Y, Hauff N, 2016a High-throughput allele-specific expression across 250 environmental conditions. Genome Res 26: 1627–1638. 27934696 10.1101/gr.209759.116 PMC5131815

[GR237354KALC40] Moyerbrailean GA, Kalita CA, Harvey CT, Wen X, Luca F, Pique-Regi R. 2016b Which genetics variants in DNase-seq footprints are more likely to alter binding? PLoS Genet 12: e1005875. 26901046 10.1371/journal.pgen.1005875 PMC4764260

[GR237354KALC41] Muerdter F, Boryń ŁM, Woodfin AR, Neumayr C, Rath M, Zabidi MA, Pagani M, Haberle V, Kazmar T, Catarino RR, 2017 Resolving systematic errors in widely used enhancer activity assays in human cells. Nat Methods 15: 141–149. 29256496 10.1038/nmeth.4534 PMC5793997

[GR237354KALC42] Nakagawa M, Shimabe M, Nishimoto N, Watanabe-Okochi N, Ichikawa M, Nannya Y, Imai Y, Kurokawa M. 2009 AML1/Runx1 is a cytoplasmic attenuator of NF-Kb signaling: implication in pathogenesis and targeted therapy of AML1-related leukemia. Blood 114: 1962.

[GR237354KALC43] Nakagawa M, Shimabe M, Watanabe-Okochi N, Arai S, Yoshimi A, Shinohara A, Nishimoto N, Kataoka K, Sato T, Kumano K, 2011 AML1/RUNX1 functions as a cytoplasmic attenuator of NF-κB signaling in the repression of myeloid tumors. Blood 118: 6626–6637. 22021368 10.1182/blood-2010-12-326710

[GR237354KALC44] Nédélec Y, Sanz J, Baharian G, Szpiech ZA, Pacis A, Dumaine A, Grenier JC, Freiman A, Sams AJ, Hebert S, 2016 Genetic ancestry and natural selection drive population differences in immune responses to pathogens. Cell 167: 657–669.e21. 27768889 10.1016/j.cell.2016.09.025

[GR237354KALC45] Ollivier V, Parry GC, Cobb RR, de Prost D, Mackman N. 1996 Elevated cyclic AMP inhibits NF-*κ*B-mediated transcription in human monocytic cells and endothelial cells. J Biol Chem 271: 20828–20835. 8702838 10.1074/jbc.271.34.20828

[GR237354KALC46] Pacis A, Tailleux L, Morin AM, Lambourne J, MacIsaac JL, Yotova V, Dumaine A, Danckaert A, Luca F, Grenier JC, 2015 Bacterial infection remodels the DNA methylation landscape of human dendritic cells. Genome Res 25: 1801–1811. 26392366 10.1101/gr.192005.115 PMC4665002

[GR237354KALC47] Papadakis KA, Zhu D, Prehn JL, Landers C, Avanesyan A, Lafkas G, Targan SR. 2005 Dominant role for TL1A/DR3 pathway in IL-12 plus IL-18-induced IFN-γ production by peripheral blood and mucosal CCR9^+^ T lymphocytes. J Immunol 174: 4985–4990. 15814728 10.4049/jimmunol.174.8.4985

[GR237354KALC48] Parry GC, Mackman N. 1997 Role of cyclic AMP response element-binding protein in cyclic AMP inhibition of NF-κB-mediated transcription. J Immunol 159: 5450–5456. 9548485

[GR237354KALC49] Patwardhan R, Hiatt J, Witten D, Kim M. 2012 Massively parallel functional dissection of mammalian enhancers *in vivo*. Nat Biotechnol 30: 265–270. 22371081 10.1038/nbt.2136 PMC3402344

[GR237354KALC50] Pickrell J. 2014 Joint analysis of functional genomic data and genome-wide association studies of 18 human traits. Am J Hum Genet 94: 559–573. 24702953 10.1016/j.ajhg.2014.03.004 PMC3980523

[GR237354KALC51] Prehn JL, Mehdizadeh S, Landers CJ, Luo X, Cha SC, Wei P, Targan SR. 2004 Potential role for TL1A, the new TNF-family member and potent costimulator of IFN-γ, in mucosal inflammation. Clin Immunol 112: 66–77. 15207783 10.1016/j.clim.2004.02.007

[GR237354KALC53] Rao SS, Huntley MH, Durand NC, Stamenova EK, Bochkov ID, Robinson JT, Sanborn AL, Machol I, Omer AD, Lander ES, 2014 A 3D map of the human genome at kilobase resolution reveals principles of chromatin looping. Cell 159: 1665–1680. 25497547 10.1016/j.cell.2014.11.021 PMC5635824

[GR237354KALC54] Sharon E, Kalma Y, Sharp A, Raveh-Sadka T. 2012 Inferring gene regulatory logic from high-throughput measurements of thousands of systematically designed promoters. Nat Biotechnol 30: 521–530. 22609971 10.1038/nbt.2205 PMC3374032

[GR237354KALC55] Smale ST. 2010 Selective transcription in response to an inflammatory stimulus. Cell 140: 833–844. 20303874 10.1016/j.cell.2010.01.037 PMC2847629

[GR237354KALC56] Smith T, Heger A, Sudbery I. 2017 UMI-tools: modeling sequencing errors in Unique Molecular Identifiers to improve quantification accuracy. Genome Res 27: 491–499. 28100584 10.1101/gr.209601.116 PMC5340976

[GR237354KALC57] Stormo GD, Zuo Z, Chang YK. 2015 Spec-seq: determining protein–DNA-binding specificity by sequencing. Brief Funct Genomic 14: 30–38. 10.1093/bfgp/elu043 PMC4366588 25362070

[GR237354KALC58] Stouffer SA, Suchman EA, Devinney LC, Star SA, Williams RMJ. 1949 The American soldier: adjustment during army life, Vol. 265, pp. 173–175. Princeton University Press, Princeton, NJ.

[GR237354KALC59] Stranger BE. 2007 Population genomics of human gene expression. Nat Genet 39: 1217–1224. 17873874 10.1038/ng2142 PMC2683249

[GR237354KALC60] Takedatsu H, Michelsen KS, Wei B, Landers CJ, Thomas LS, Dhall D, Braun J, Targan SR. 2008 TL1A (TNFSF15) regulates the development of chronic colitis by modulating both T-helper 1 and T-helper 17 activation. Gastroenterology 135: 552–567. 18598698 10.1053/j.gastro.2008.04.037 PMC2605110

[GR237354KALC61] Tewhey R, Kotliar D, Park D, Liu B, Winnicki S, Reilly S, Andersen K, Mikkelsen T, Lander E, Schaffner S, 2016 Direct identification of hundreds of expression-modulating variants using a multiplexed reporter assay. Cell 165: 1519–1529. 27259153 10.1016/j.cell.2016.04.027 PMC4957403

[GR237354KALC62] Ulirsch J, Nandakumar S, Wang L, Giani F, Zhang X, Rogov P, Melnikov A, McDonel P, Do R, Mikkelsen T, 2016 Systematic functional dissection of common genetic variation affecting red blood cell traits. Cell 165: 1530–1545. 27259154 10.1016/j.cell.2016.04.048 PMC4893171

[GR237354KALC63] Vanhille L, Griffon A, Maqbool MA, Zacarias-Cabeza J, Dao LTM, Fernandez N, Ballester B, Andrau JC, Spicuglia S. 2015 High-throughput and quantitative assessment of enhancer activity in mammals by CapStarr-seq. Nat Commun 6: 6905. 25872643 10.1038/ncomms7905

[GR237354KALC64] Vietri Rudan M, Barrington C, Henderson S, Ernst C, Odom D, Tanay A, Hadjur S. 2015 Comparative Hi-C reveals that CTCF underlies evolution of chromosomal domain architecture. Cell Rep 10: 1297–1309. 25732821 10.1016/j.celrep.2015.02.004 PMC4542312

[GR237354KALC65] Vockley C, Guo C, Majoros W. 2015 Massively parallel quantification of the regulatory effects of noncoding genetic variation in a human cohort. Genome Res 25: 1206–1214. 26084464 10.1101/gr.190090.115 PMC4510004

[GR237354KALC66] Wang X, He L, Goggin S, Saadat A, Wang L, Claussnitzer M, Kellis M. 2017 High-resolution genome-wide functional dissection of transcriptional regulatory regions in human. bioRxiv doi: 10.1101/193136. PMC6300699 30568279

[GR237354KALC67] Wen X, Luca F, Pique-Regi R. 2015 Cross-population joint analysis of eQTLs: fine mapping and functional annotation. PLoS Genet 11: e1005176. 25906321 10.1371/journal.pgen.1005176 PMC4408026

[GR237354KALC68] Wong D, Teixeira A, Oikonomopoulos S, Humburg P, Lone I, Saliba D, Siggers T, Bulyk M, Angelov D, Dimitrov S, 2011 Extensive characterization of NF-κB binding uncovers non-canonical motifs and advances the interpretation of genetic functional traits. Genome Biol 12: R70. 21801342 10.1186/gb-2011-12-7-r70 PMC3218832

[GR237354KALC69] Yamazaki K, McGovern D, Ragoussis J, Paolucci M, Butler H, Jewell D, Cardon L, Takazoe M, Tanaka T, Ichimori T, 2005 Single nucleotide polymorphisms in *TNFSF15* confer susceptibility to Crohn's disease. Hum Mol Genet 14: 3499–3506. 16221758 10.1093/hmg/ddi379

[GR237354KALC70] Zhao B, Barrera LA, Ersing I, Willox B, Schmidt SC, Greenfeld H, Zhou H, Mollo SB, Shi TT, Takasaki K, 2014 The NF-κB genomic landscape in lymphoblastoid B cells. Cell Rep 8: 1595–1606. 25159142 10.1016/j.celrep.2014.07.037 PMC4163118

