# Supplementary material for: High-throughput characterization of genetic effects on DNA–protein binding and gene transcription
Source: Genome Res. 2018 Nov;28(11):1701–8. doi: 10.1101/gr.237354.118 (PMC6211638; doi:10.1101/gr.237354.118)
Supplement: Supplemental Material [file supp_28_11_1701__index.html]

High-throughput characterization of genetic effects on DNA–protein binding and gene transcription — Supplemental Material 

# High-throughput characterization of genetic effects on DNA–protein binding and gene transcription

## Supplemental Material

- supplement\_tex.pdf
- Supplemental\_Fig\_S1.png
- Supplemental\_Fig\_S2.png
- Supplemental\_Fig\_S3.png
- Supplemental\_Fig\_S4.png
- Supplemental\_Fig\_S5.png
- Supplemental\_Fig\_S6.png
- Supplemental\_Fig\_S7.png
- Supplemental\_Fig\_S8.png
- Supplemental\_Fig\_S9.pdf
- Supplemental\_Fig\_S10.png
- Supplemental\_Methods\_S2.pdf
- Supplemental\_Table\_S1.txt
- Supplemental\_Table\_S2.txt
- Supplemental\_Table\_S3.txt
- Supplemental\_Table\_S4.txt
- Supplemental\_Table\_S7.txt
- Supplemental\_Table\_S8.txt
- Supplemental\_Table\_S9.txt
- Supplemental\_Table\_S10.txt
- Supplemental\_Table\_S11.txt
- Supplemental\_Table\_S14.txt
- Supplemental\_Table\_S15.txt
- Supplemental\_Methods\_dna.zip
- supplement\_texfile.zip
