# Supplementary material for: High-throughput characterization of genetic effects on DNA–protein binding and gene transcription
Source: Genome Res. 2018 Nov;28(11):1701–8. doi: 10.1101/gr.237354.118 (PMC6211638; doi:10.1101/gr.237354.118)
Supplement: Supplemental Material [file supp_gr.237354.118_supplement_tex.pdf]

High throughput characterization of genetic effects on DNA:protein binding and gene transcription

Cynthia A. Kalita [1], Christopher D. Brown [2], Andrew Freiman [1],
Jenna Isherwood [1], Xiaoquan Wen[3], Roger Pique-Regi [1,4,*], Francesca Luca [1,4,*]

[1]Center for Molecular Medicine and Genetics, Wayne State University
[2]Department of Genetics, University of Pennsylvania
[3]Department of Biostatistics, University of Michigan
[4]Department of Obstetrics and Gynecology, Wayne State University

[*]To whom correspondence should be addressed: rpique@wayne.edu, fluca@wayne.edu.

Supplementary Methods

Oligo selection and design

Tables S2, S3, S4 report the annotations we have considered with their sources and Table (S16, S12) includes the library composition. These included: SNPs predicted to alter transcription factor binding in LCLs and HepG2 (CentiSNPs, (Moyerbrailean et al. 2015)), LCL eQTLs fine-mapped in (Wen et al. 2015), liver eQTLs (Innocenti et al. 2011), significant fgwas SNPs in transcription factor binding motifs for 18 complex traits (Moyerbrailean et al. 2016b), significant fgwas SNPs for base models of functional annotations for 18 complex traits (Pickrell 2014), ASB SNPs, and strong enhancers with no predicted ASB (regions with footprints containing SNPs that are not predicted to affect binding of TFs) (Moyerbrailean et al. 2015). CentiSNP is an annotation that we recently developed (Moyerbrailean et al. 2016b), and that uses the CENTIPEDE framework (Pique-Regi et al. 2011) to integrate DNase-seq footprints with a recalibrated position weight matrix (PWM) model for the sequence to predict the functional impact of SNPs in footprints. SNPs in footprints "footprint-SNPs" are further categorized using CENTIPEDE hierarchical prior for each allele as "CentiSNP" if the prior relative odds for binding are >20. FASTA sequences with a window of 99 (on each side of the SNP) on the BED file were grabbed using seqBedFor2bit (Moyerbrailean et al. 2015), and 15bp matching sequencing primers used for Illumina NGS were added to each end. This generates an oligo containing 200bp of regulatory region with the SNP centered in the middle, with primers on both ends (Figure S1). Each regulatory region was designed to have two oligos: one for each of the alleles. A second list of the FASTA sequences without the primer ends was generated to use as a custom reference genome, then converted to fastq using fatofastq (UCSC genomics utilities). The full SNP list was aligned to the hg19 genome with BWA mem (Li 2013), removing the regions that aligned with a quality score less than 20 (unique alignment probability $>$ 99%). The full SNP list was also aligned to the custom reference genome, and then filtered for a quality score of 190. A total of 39,366 indexes were randomly generated to match this pattern: RDHBVDHBVD. This sequence was chosen to limit the longest possible polyACGT run at any position to 3 nucleotides, and avoid a G in the first and last position (corresponding to a dark cycle on the Illumina NextSeq 500).

Oligo synthesis and amplification

DNA inserts 230bp long, corresponding to 200bp of regulatory sequence, were synthesized by Agilent to contain the regulatory region and the SNP of interest within the first 150bp. We performed a first round of PCR using Phusion High-Fidelity PCR Master Mix with HF Buffer (NEB) and primers [F_transposase and R_transposase] (Table S17) with cycling conditions: 98°C for 30s, followed by 4 cycles of 98°C for 10s, 50°C for 30s, 72°C for 60s, followed by 6 cycles of 98°C for 10s, 65°C for 30s, 72°C for 60s, followed by 72°C for 5 min. This reaction was used to double strand the oligos and complete the sequencing primers. The PCR product was run on a 2% agarose gel, extracted and purified with the NucleoSpin Gel and PCR Clean-Up Kit (Clontech). A subsequent round of PCR amplified the material using the same reaction

as in the first round of PCR, but with cycling conditions: 98°C for 30s, followed by 15 cycles of 98°C for 10s, 65°C for 30s, 72°C for 60s, followed by 72°C for 5min. The PCR product was purified as described above.

Cloning Regulatory regions into pGL4.23

A recent study demonstrated that the ORI can be an active promoter in pGL4.23 plasmids and can function as a stronger promoter in the absence of other promoter sequences (Muerdter et al. 2017). Here we used a design that includes a minimal promoter, thus potentially missing some signal from the weakest enhancer sequences in our library. However, as we focus on allele-specific enhancer activity, the presence of a minimal promoter in addition to the ORI, should affect both alleles similarly and should not induce false positives in the allele-specific signal. Plasmid pGL4.23 (Promega) was linearized using CloneAmp HiFi PCR Premix (Clontech), primers [STARR_F_SH and STARR_R_SH], and 35 cycles of 98°C for 10s, 60°C for 15s, and 72°C for 5s. The PCR product was purified on a 1% agarose gel as described above. Inserts were cloned into the linear plasmid using standard Infusion (Clontech) cloning protocol. Clones (Supplemental methods: BiT-STARR-seq plasmid) were transformed into XL10-Gold Ultracompetent Cells (Agilent) in a total of 7 reactions. These reactions were pooled and grown overnight in 500ml LB at 37°C in a shaking incubator. DNA was extracted using Endofree maxiprep kit (Qiagen).

Transfection of library

Previous studies (Muerdter et al. 2017; Huerfano et al. 2013) have found that transfection, especially from nucleofection, can lead to activation of type 1 interferon response, which may complicate comparison of enhancer activities between different cell types. In our study design, allelic effects are measured and contrasted within the same cell type, thus any trans-effect is inherently controlled. Furthermore, in LCLs the immune response is already activated because of EBV transformation. DNA library was transfected into LCLs (GM18507) using standard nucleofection protocol, program DS150, 3μg of DNA and 7.5×10^6 cells. A total of 3 sets of transfections were done in triplicate cuvettes, then pooled. We performed nine biological replicates of the transfection from 7 independent cell growth cultures. After transfection, cells were incubated at 37°C and 5% CO2 in RPMI1640 with 15%FBS and 1% Gentamycin for 24h. Cell pellets were then lysed using RLT lysis buffer (Qiagen), and cryopreserved at -80°C.

Library preparation

RNA-libraries. Thawed lysates were split in three aliquotes and total RNA was isolated using RNeasy Plus Mini Kit (Qiagen). Poly-adenylated RNA was selected using Dynabeads mRNA Direct Kit (Ambion) using the protocol for total RNA input. RNA was reverse transcribed to cDNA using Superscript III First-Strand Synthesis kit (ThermoFisher) with primer [Nextera_i7_10N] and following the manufacturer's protocol. cDNA technical replicates were pooled and SPRI Select beads (Life Tech) were used for purification and size selection at a ratio of 0.9X. PCR Library Enrichment was performed using a nested PCR protocol. For the first round of PCR we used Phusion High-Fidelity PCR Master Mix with HF Buffer (NEB) and

primers [F_trans_short and Illumina2.1] with cycling conditions: 98°C for 30s, followed by 15 cycles of 98°C for 10s, 72°C for 15s, followed by 72°C for 5 min. PCR product was purified on a 2% agarose gel as described above. The nested PCR used Phusion High-Fidelity PCR Master Mix with HF Buffer (NEB) and primers [fixed N5xx adapter (Illumina) (unique per each library replicate) and Illumina2.1] with cycling conditions: 98°C for 30s, followed by 5 cycles of 98°C for 10s, 72°C for 15s, followed by 72°C for 5 min. In a side quantitative real-time PCR reaction, 5μL of PCR product, 10X SYBR Green I, and the same primers and master mix were run in conditions: 98°C for 30s, 30 cycles of 98°C for 10s, 63°C for 30s, and 72°C for 60s. To determine the number of PCR cycles needed to reach saturation, we plotted linear Rn versus cycle and determined the cycle number that corresponds to 25% of maximum fluorescent intensity on the side reaction (Buenrostro et al. 2013). The PCR product (Figure S1) was purified on a 2% agarose gel as described above.

DNA-libraries. We prepared 7 replicates of the DNA library using the PCR protocol as described in (Buenrostro et al. 2013) except using primers [fixed N5xx adapter (Illumina) (unique per each library replicate) and Nextera_i7_10N] and 30ng of input plasmid DNA. PCR product was purified on a 2% agarose gel as described above.

BiT-BUNDLE-seq

We used BiT-BUNDLE-seq, a new version of the BUNDLE-seq protocol (Levo et al. 2015). Input DNA sequences were extracted from the BiT-STARR-seq DNA plasmid library using the same PCR conditions as in preparing the DNA libraries, followed by purification on a 2% agarose gel as described above. We used N-terminal GST-tagged, recombinant human NFKB1 from EMD Millipore. The reaction buffer (0.15 M NaCl, 0.5 mM PMSF [Sigma], 1 mM BZA [Sigma], 0.5X TE, and 0.16 μg/μL PGA [Sigma]) was incubated at room temperature for 2 hours in low binding tubes (ThermoFisher). The tubes were cooled for 30 min at 4°C, and then 0.067 μg/μL BSA (Sigma) was added before adding the NFKB1 protein. One hundred nanograms of DNA were then added, and the protein and DNA were incubated for 1 h at 4°C. Experiments were performed in triplicates for each NFKB1 concentration.

The reaction mix was run with 6μL Ficoll (Sigma) in a 7.5% Mini-PROTEAN TGX Precast 10-well Protein Gel (BIORAD) in cold 0.25X TBE buffer for 2 hours at 100V. The gel was stained for 30 min with 3X GelStar (Lonza). Bound and unbound DNA bands were excised under a blue light transilluminator. The DNA was eluted from the gel using the QIAQuick Gel Extraction Kit with a User-Developed Protocol (Qiagen QQ05). The gel slices were incubated in a diffusion buffer (0.5 M ammonium acetate, 10mM magnesium acetate, 1mM EDTA, ph 8.0 [KD Medical]; 0.1% SDS [Sigma]) at 50°C for 30 minutes. The supernatant was then passed through a disposable plastic column containing packed, siliconized glass wool [Supelco] to remove any residual polyacrylamide. Libraries were then quantified and loaded on the NextSeq 500 for sequencing.

Library Sequencing

Pooled RNA and DNA libraries were sequenced on the Illumina Nextseq 500 to generate 125 cycles for read 1, 30 cycles for read 2, 8 cycles for the fixed multiplexing index 2 and 10

cycles for index 1 (variable barcode). Sequencing depth for each replicate can be found in Table S18.

Data Processing

Reads were mapped using the HISAT2 aligner (Kim et al. 2015), using the 1Kgenomes snp index so as to avoid reference bias. First we removed variants whose UMI was not possible to be present, given the UMI pattern selected. We then ran UMItools (Smith et al. 2017) using standard flags, as well as a q20 filter. We then ran the deduplicated files through mpileup using a BED file of our full SNP list, the -t DP4, -g, and -d 1000000. DNA reads were processed through a counts filter (on the summed replicates) of more than 7 counts per SNP and at least one count for the reference and alternate alleles in either direction. 50,609 SNPs in the DNA library were used as input to the RNA library. The RNA library was processed following the same procedure as for the DNA library, except that the counts filter required a count of >1 per SNP and at least one count for both reference and alternate alleles. To identify SNPs with allele-specific effects, we applied QuASAR-MPRA (Kalita et al. 2017), where for each SNP the reference and alternate allele counts were compared to the DNA proportion. QuASAR-MPRA results from each replicate were then combined using the fixed effects method, and corrected for multiple tests using BH procedure (Benjamini Hochberg 1995). The effect size $\beta_{l,n}$ of each replicate n is weighted by $w_{n,l} = 1/\hat{\sigma}^2_{n,l}$, to calculate the overall effect size and standard error:

$$\beta_l^* = \frac{1}{w_l^*} \sum_n \beta_{n,l}\, w_{n,l} \qquad\qquad \sigma_l^* = \sqrt{1/w_l^*} \tag{1}$$

where $w_l^* = \sum_n w_n, l$. We can then calculate the Z-score and p-value to test for an overall change between all the RNA replicates combined with respect to the original DNA proportion β_0:

$$Z_l = \frac{\beta_l^* - \beta_0}{\sigma_l^*}\,, \beta_0 = \log \frac{r_l}{1 - r_l}\,, \qquad\qquad p = 2\Phi(-|Z_l|) \tag{2}$$

We used the genomic inflation test to calculate the genomic inflation parameter, λ, for a set of p-values (Yang et al. 2011). For this we calculated the ratio of the median of the p-value distribution to the expected median, thus quantifying the extent of the bulk inflation and the excess false positive rate.

BiT-BUNDLE-seq data analysis

Counts from both the unbound and bound DNA were combined, and a filter was set so that each SNP direction combination had 5 counts for each allele. This combined count was also used to calculate a reference proportion. Each replicate for the bound and unbound libraries were then run through QuASAR-MPRA using the calculated reference proportion. These were then compared using ΔAST (Moyerbrailean et al. 2016a) to identify ASB in the bound fraction that is differential relative to the unbound fraction. The replicates were combined using Stouffers method (STOUFFER et al. 1949) to identify ASB for each NFKB1 concentration, and

combined again to identify the total ASB. The unbound and bound libraries counts were additionally analyzed with DESeq2 (Love et al. 2014) to identify over-represented bound enhancer regions (FDR 1% and logFC>1). To better estimate the dispersion parameters, the DESeq2 model was fit on all sequencing data and without merging the replicate libraries:

$$K_{ij} \sim \text{NB}(\mu_{ij}, \alpha_i) \tag{3}$$

$$\mu_{ij} = s_j q_{ij} \tag{4}$$

$$\log_2(q_{ij}) = \beta_{i,0} + \beta_{i,\text{C}(j)} + \beta_{i,\text{B}(j)} \tag{5}$$

For each enhancer region i and sample j, the read counts K_{ij} are modeled using a negative binomial distribution with fitted mean μ_{ij} and an enhancer region-specific dispersion parameter α_i. The fitted mean is composed of a sample-specific size factor s_j and a parameter q_{ij} proportional to the expected true concentration of regions for sample j. The coefficient β_0 represents the mean effect intercept, $\beta_{\text{C}(j)}$ represents the lane (NFKB1 concentration:replicate) effect, and and $\beta_{\text{B}(j)}$ represents the Bound/Unbound effect for each NFKB1 concentration (High, Medium, and Low).

We then contrasted the bound to the unbound for each concentration (i.e., high concentration bound to high concentration unbound) using the default DESeq2 Wald test for each enhancer region $\beta_{\text{B}(j)} \neq= 0$, and a Benjamini-Hochberg (BH) adjusted p-value was calculated with automatic independent filtering (DESeq2 default setting).

GWAS overlap

SNPs nominally significant (p<0.05) for ASB (identified with ΔAST) or ASE (identified with QuASAR-MPRA) that were also annotated as CentiSNP were overlapped with SNPs from the GWAS catalogue (V6) (MacArthur et al. 2017), as well as with SNPs fine-mapped with the fgwas software as in (Moyerbrailean et al. 2016b).

BiT-STARR-seq plasmid

pGL4.23 plasmid with an example cloned in insert.
`file://Supplemental_Methods_S1.dna`

BiT-STARR-seq Protocol

`file://Supplemental_Methods_S2.pdf`

Supplementary Tables

Table 1: **BiT-STARR-seq results**. QuASAR-MPRA results for BiT-STARR-seq.

`file://Supplemental_Table_S1.txt`

Table 2: **Annotations Used: CentiSNPs**. SNP annotations used for overlap with BiT-BUNDLE-seq and BiT-STARR-seq. First 4 columns are in the same order for each file (chr, pos, pos1, rsID). Column 5 contains the transcription factor with a CentiSNP at that location.

file://Supplemental_Table_S2.txt

Table 3: **Annotations Used: GWAS**. SNP annotations used for overlap with BiT-BUNDLE-seq and BiT-STARR-seq. First 4 columns are in the same order for each file (chr, pos, pos1, rsID). Column 5 contains the GWAS trait associated with the SNP.

file://Supplemental_Table_S3.txt

Table 4: **Annotations Used: eQTL**. SNP annotations used for overlap with BiT-BUNDLE-seq and BiT-STARR-seq. First 4 columns are in the same order for each file (chr, pos, pos1, rsID). eQTL SNPs. Column 5 contains the information for whether the eQTL was identified in cells infected with L (*Listeria*), S (*Salmonella*), or NI (not infected). Column 6 contains the gene associated with the eQTL. Column 7 contains the beta for the eQTL association. Column 8 contains the p-value for the eQTL association.

file://Supplemental_Table_S4.txt

Table 5: **Distribution of ASE Z scores**. For each regulatory category: KS-test results from comparing the Z score distribution for ASE for the category vs the negative control.

Reg Cat	p-value
CentiSNPs	2.44×10^{-6}
ASH	4.28×10^{-4}
Liver_eQTLs	3.19×10^{-4}
LCL_eQTLs	0.01
fgwas_SNPs	$<2.20 \times 10^{-16}$

Table 6: **Transcription factors in BiT-STARR-seq**. Number of SNPs in motifs matching the top 10 covered transcription factors in BiT-STARR-seq.

Transcription Factor	Freq
CTCF	4911
E2F-1	2794
E2F	4407
ATF	5567
AML1	3794
ATF2:c-Jun	3651
CREB	12955
AP1	2673
ARG RI	3445
STF1	3561

Table 7: **DEseq results: Combined concentrations**. Differentially bound regions for combined concentrations. Columns are (identifier(rsID_Direction), adjusted p-value, p-value, logFC).

`file://Supplemental_Table_S7.txt`

Table 8: **DEseq results: Low concentration**. Differentially bound regions for low concentration. Columns are (identifier(rsID_Direction), adjusted p-value, p-value, logFC).

`file://Supplemental_Table_S8.txt`

Table 9: **DEseq results: Mid**. Differentially bound regions for mid concentration. Columns are (identifier(rsID_Direction), adjusted p-value, p-value, logFC).

`file://Supplemental_Table_S9.txt`

Table 10: **DEseq results: High**. Differentially bound regions for high concentration. Columns are (identifier(rsID_Direction), adjusted p-value, p-value, logFC).

`file://Supplemental_Table_S10.txt`

Table 11: **BiT-BUNDLE-seq results**. ΔAST results for BiT-BUNDLE-seq. Columns are identifier, z score, p-value, adjusted p-value, rsID.

`file://Supplemental_Table_S11.txt`

Table 12: **Designed Regulatory Category Content**. For each regulatory category: the number of constructs and how many were significant (FDR 10%) for ASB.

Reg Cat	Tested ASB	ASB
ASH	180	4
CentiSNPs	50359	1514
fgwas_SNPs	5811	285
Negative_Control	1676	43
LCL_eQTLs	2753	73
Liver_eQTLs	29070	1009

Table 13: **Enrichment for ASB and ASE variants in TFs**. For each transcription factor: enrichment results from a Fishers test for ASE in the category vs being in the TF, subset for having ASB.

TF	OR	p-value
AML1	4.61	0.01
CREB1	Inf	0.02
CTCF	2.86	0.07
CREB	1.31	0.35
ARG RI	1.37	0.58
STF1	1.26	0.83
E2F	0.98	0.84
ATF	0.94	0.86
AP1	1.21	1.00
ATF:c-Jun	1.10	1.00

Table 14: **ASB and complex traits**. ΔAST results for BiT-BUNDLE-seq. SNPs are nominally significant, associated to a complex trait, and are also CentiSNPs. Columns are rsID, direction, p-value, complex trait.

`file://Supplemental_Table_S14.txt`

Table 15: **ASE and complex traits**. QuASAR-MPRA results for BiT-STARR-seq. SNPs are nominally significant, associated to a complex trait, and are also CentiSNPs. Columns are rsID, direction, p-value, complex trait.

`file://Supplemental_Table_S15.txt`

Table 16: **Designed Regulatory Category Content**. For each regulatory category: the number of constructs and how many were significant (FDR 10%) for ASE and enrichment results from a Fishers test for ASE in the category vs the negative control.

Reg Cat	Tested ASE	Sig ASE	OR ASE	p-value ASE
ASH	162	5	1.77	0.23
CentiSNPs	43615	1806	2.40	1.28×10^{-5}
fgwas_SNPs	4894	338	4.12	1.54×10^{-13}
Negative_Control	1111	20	NA	NA
LCL_eQTLs	2307	94	2.36	2.41×10^{-4}
Liver_eQTLs	22943	827	2.08	4.88×10^{-4}

Table 17: **Primers used in BiT-STARR-seq**

Primer	Sequence
STARR_F_SH	CCGAGCCCACGAGACCTAGAGTCGGGGCGGCCG
STARR_R_SH	TGACGCTGCCGACGAAATTATTACACGGCGATC
F_transposase	TCGTCGGCAGCGTCAGATGTGTATAAGAGACAG
R_transposase	GTCTCGTGGGCTCGGAGATGTGTATAAGAGACAG
F_trans_short	TCGTCGGCAGCGTCAGAT
I2.1	CAAGCAGAAGACGGCATACGA
Nextera_i7_10N	CAAGCAGAAGACGGCATACGAGAT**RDHBVDHBVD**GTCTCGTGGGCTCGG

.

Table 18: **Sequencing Depth**. Sequencing depth for each BiT-STARR-seq replicate. Seq depth is total reads, and after deduplication is the number of reads after removing duplicates using UMI tools.

Rep	Seq Depth	UMI Depth
Rep1	89,360,505	482,117
Rep2	55,819,932	182,865
Rep3	32,784,823	1,487,089
Rep4	34,141,541	577,343
Rep5	71,090,681	464,647
Rep6	36,835,814	987,771
Rep7	74,991,057	550,080
Rep8	61,014,562	640,360
Rep9	31,142,602	404,739

Supplemental Figures

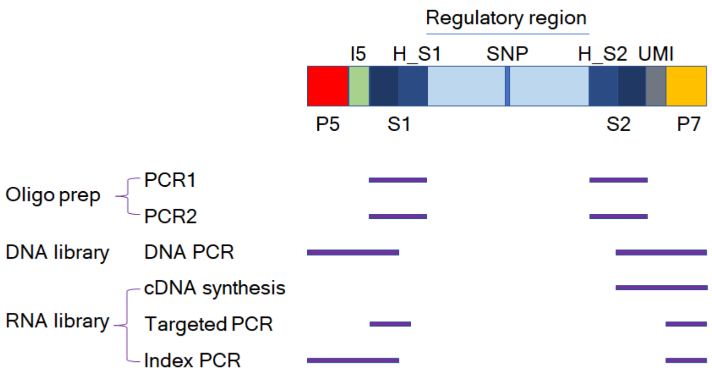

Name	Details	Length	Seq
H_S1	Seq primer handle	15	GTGTATAAGAGACAG
H_S2	Seq primer handle	15	CTGTCTCTTATACAC
S1	Seq primer	33	TCGTCGGCAGCGTCAGATGTGTATAAGAGACAG
S2	Seq primer	34	CTGTCTCTTATACACATCTCCGAGCCCACGAGAC
I5	Index	8	NNNNNNNN
UMI	UMI	10	RDHBVDHBVD
P5	Adapter	27	AATGATACGGCGACCACCGAGATCTACAC
P7	Adapter	24	ATCTCGTATGCCGTCTTCTGCTTG

Figure 1: **Schematic of oligos in BiT-STARR-seq and BiT-BUNDLE-seq**.

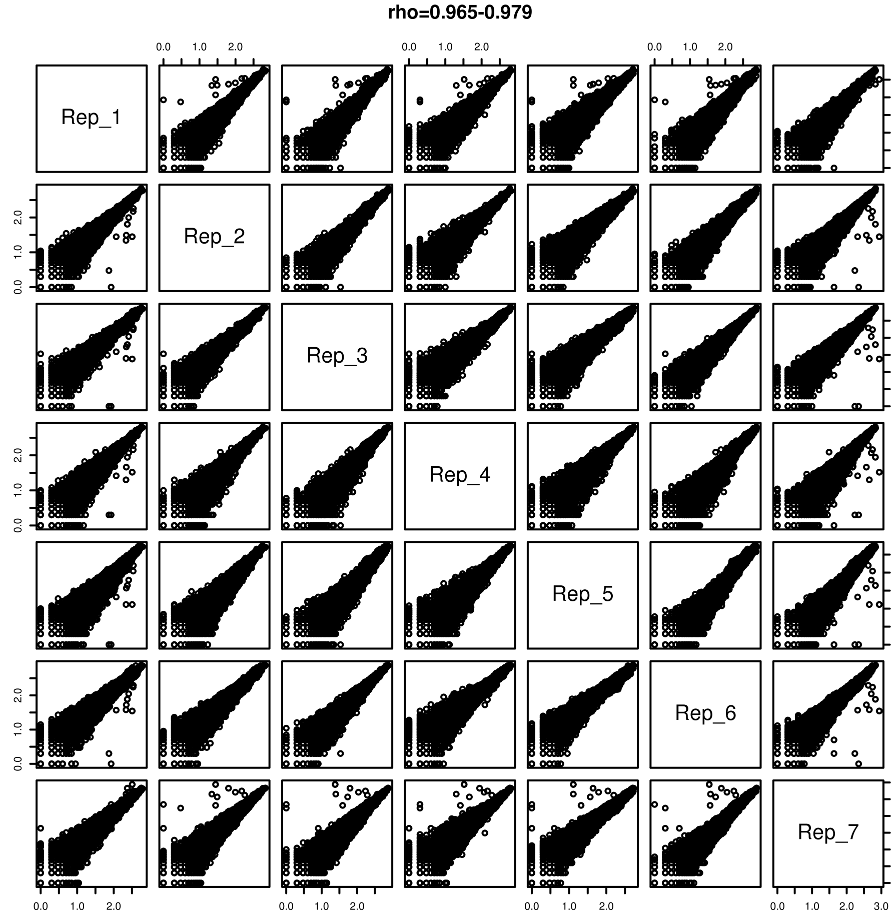

Figure 2: **Correlation of DNA libraries**. Scatterplot of filtered DNA library counts for each replicate plotted against all other replicates in $\log_{10}$ scale. Spearman rho correlation range is stated at the top.

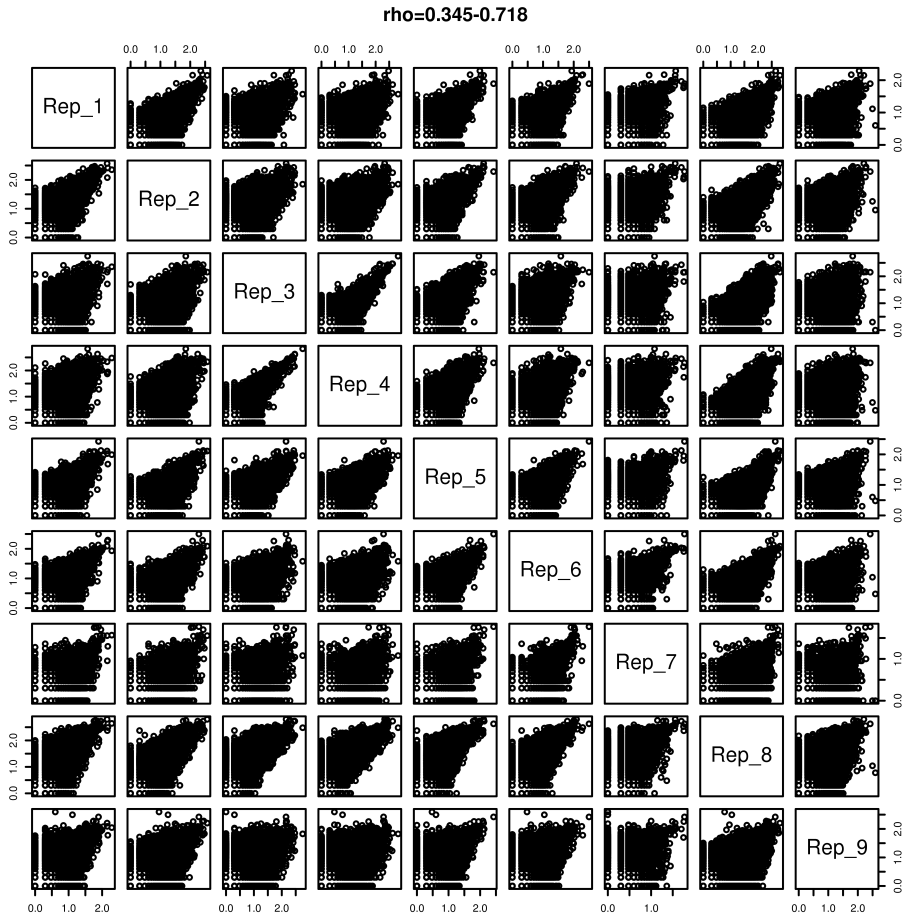

Figure 3: **Correlation of RNA libraries**. Scatterplot of filtered RNA library counts for each replicate plotted against all other replicates in $\log_{10}$ scale. Spearman rho correlation range is stated at the top.

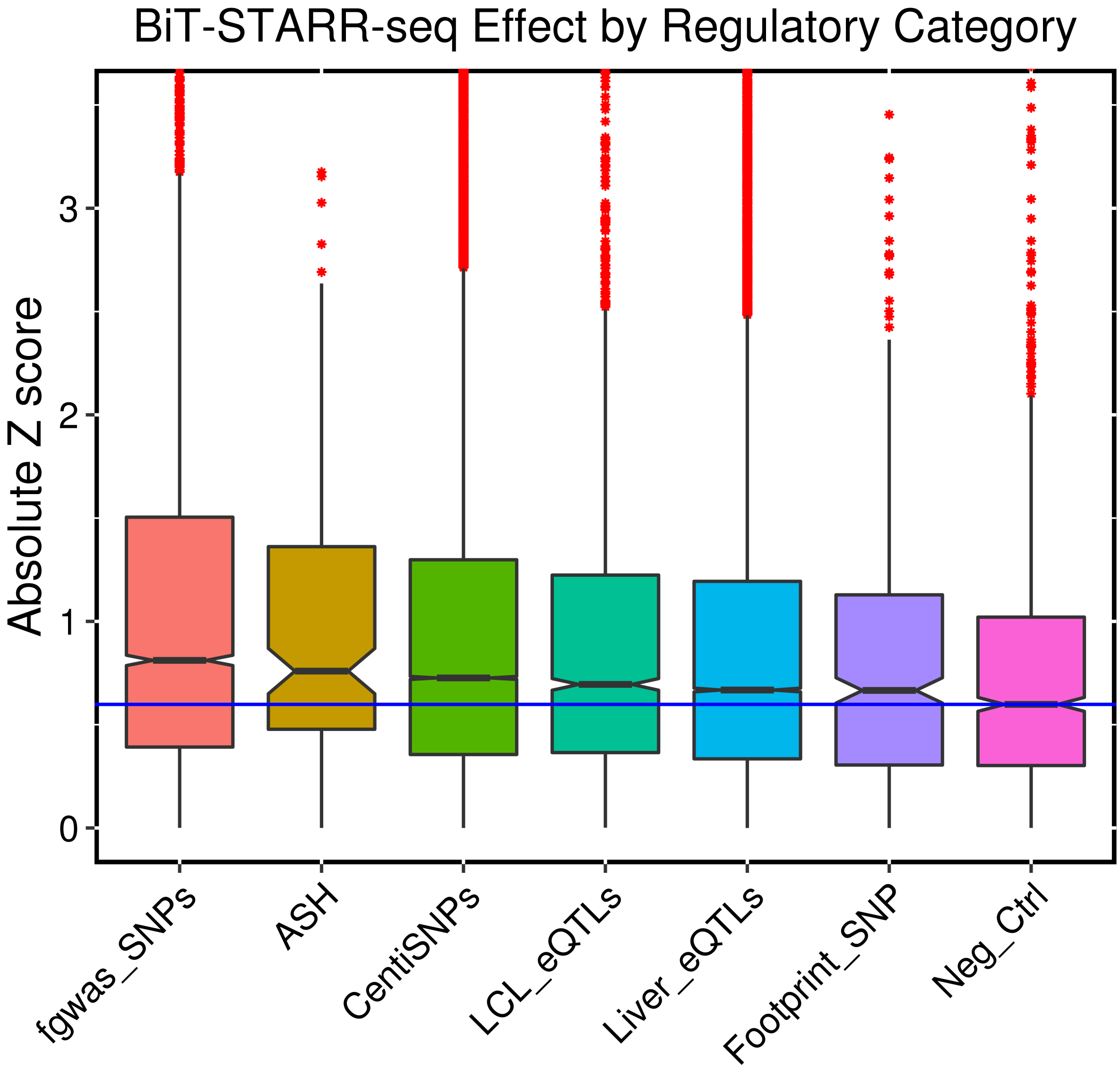

Figure 4: **BiT-STARR-seq effect by regulatory category**. Z score distribution for SNPs in each designed regulatory category. Absolute z score (y axis) for each regulatory category (x axis) is shown in the boxplot, center line of the boxplot is the median.

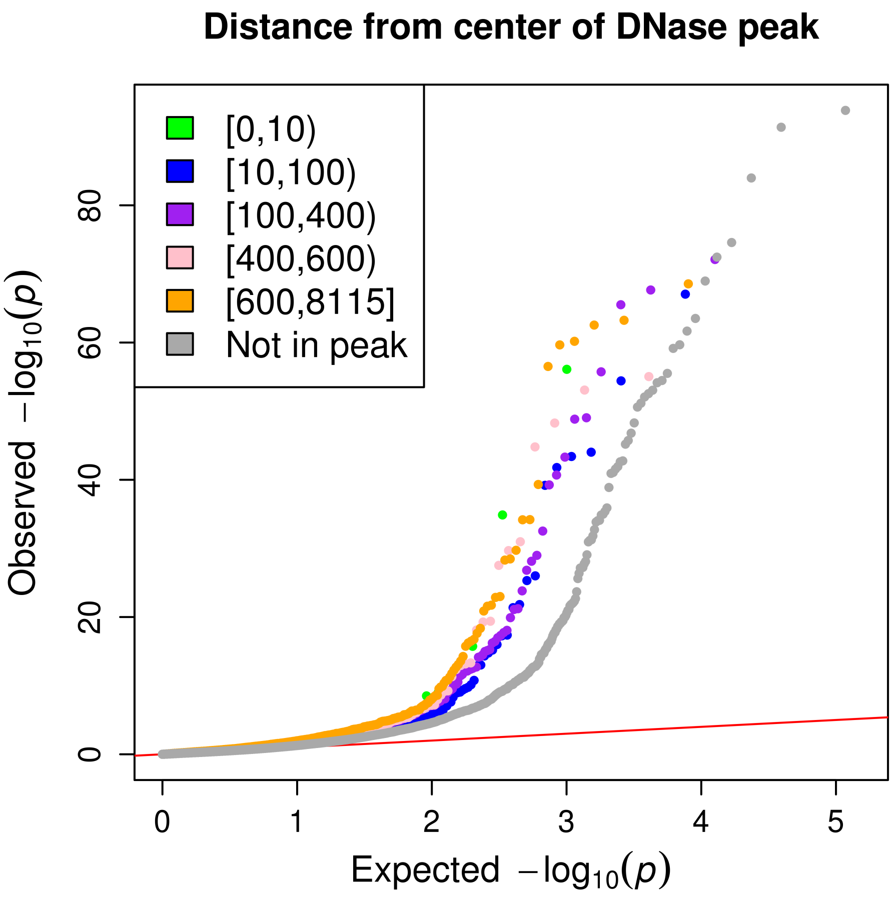

Figure 5: **DNase window centering of BiT-STARR-seq variants**. QQplot depicting the p-value distributions from QuASAR-MPRA based on how far the regulatory variant is from the center of the DNase window.

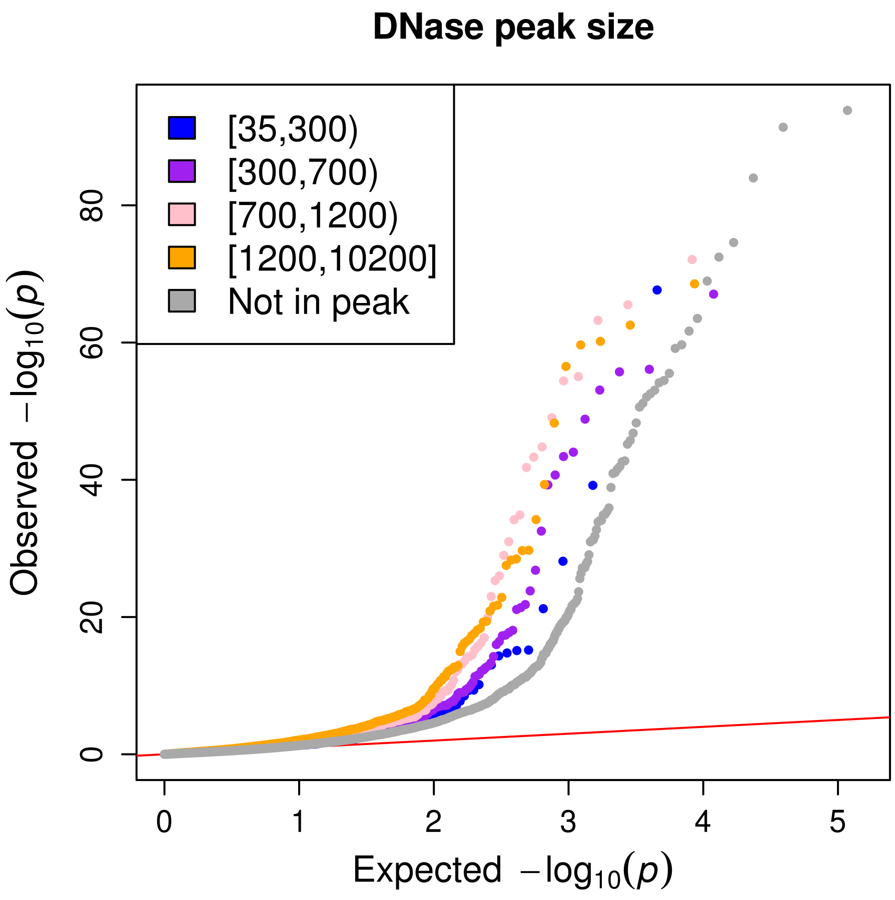

Figure 6: **DNase peak size of BiT-STARR-seq variants**. QQplot depicting the p-value distributions from QuASAR-MPRA based on the DNase peak size.

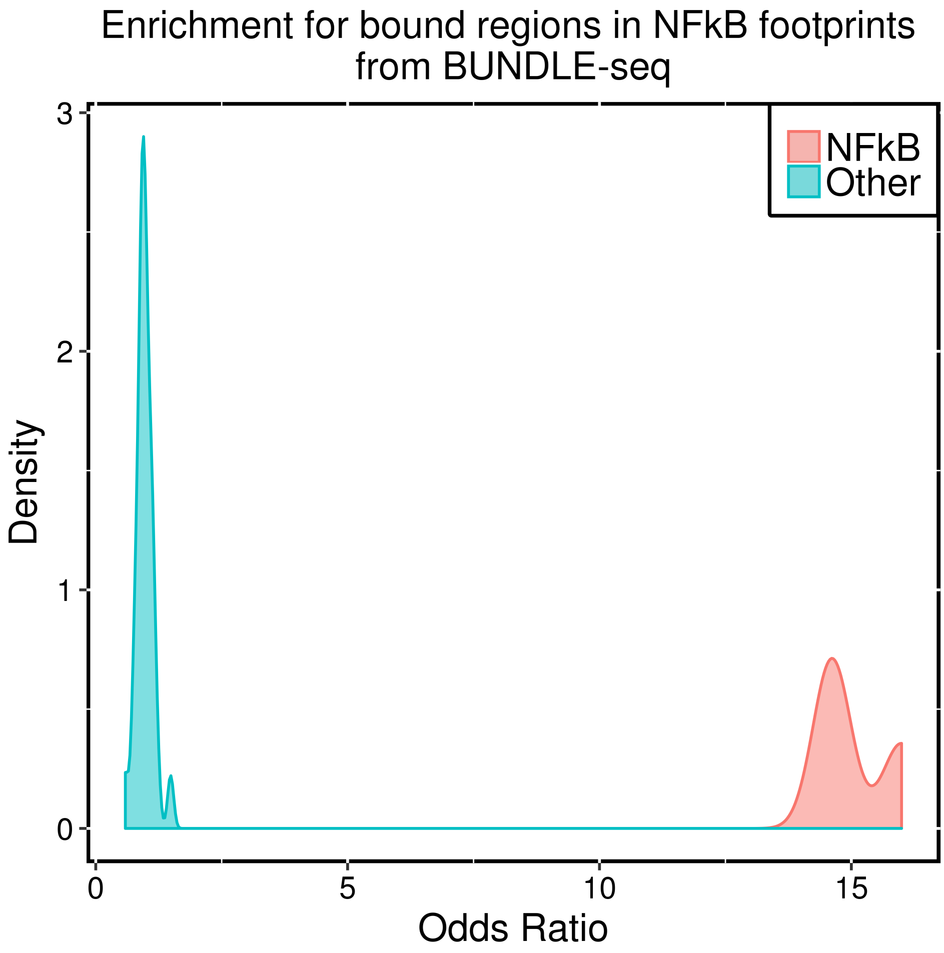

Figure 7: **Enrichment of NF-kB complex footprints in BiT-BUNDLE-seq bound regions**. Fisher′s exact test was performed to identify enrichment (x axis is the OR) for significant differentially bound regions (logFC>1 and FDR<1%). In red are the regions containing a SNP in a NF-kB complex footprint, in blue the regions containing a SNP in footprints for other transcription factors.

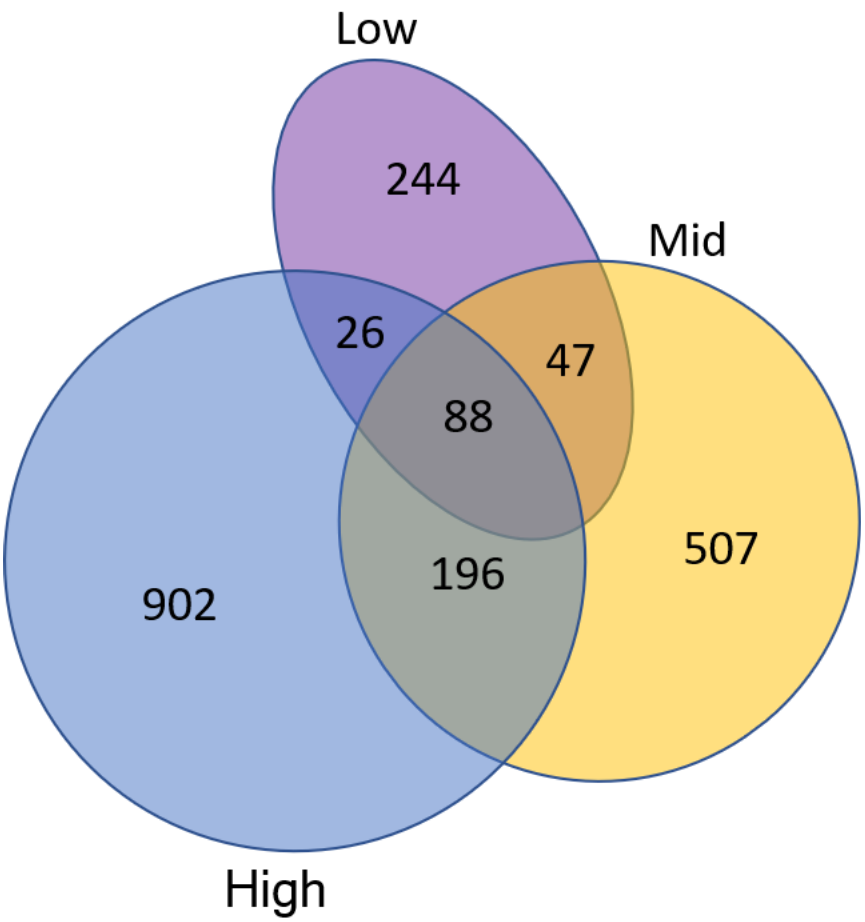

Figure 8: **Overlap between constructs with significant ASB for each concentration in BiT-BUNDLE-seq**. In purple are the number ASB at low concentration, in yellow are the number ASB at mid concentration, and in blue are the number ASB at high concentration.

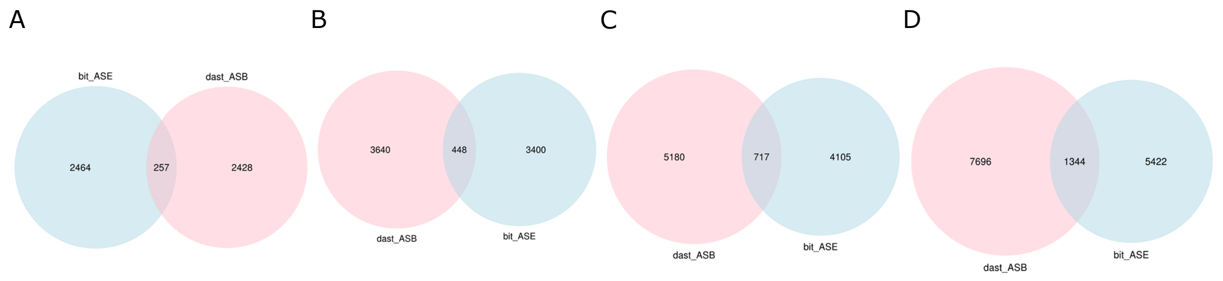

Figure 9: **Overlap between constructs with significant ASB or ASE**. A) Overlap at 10% FDR. B) Overlap at 20% FDR. C) Overlap at 30% FDR. D) Overlap at nominal p-value (p-value<0.05).

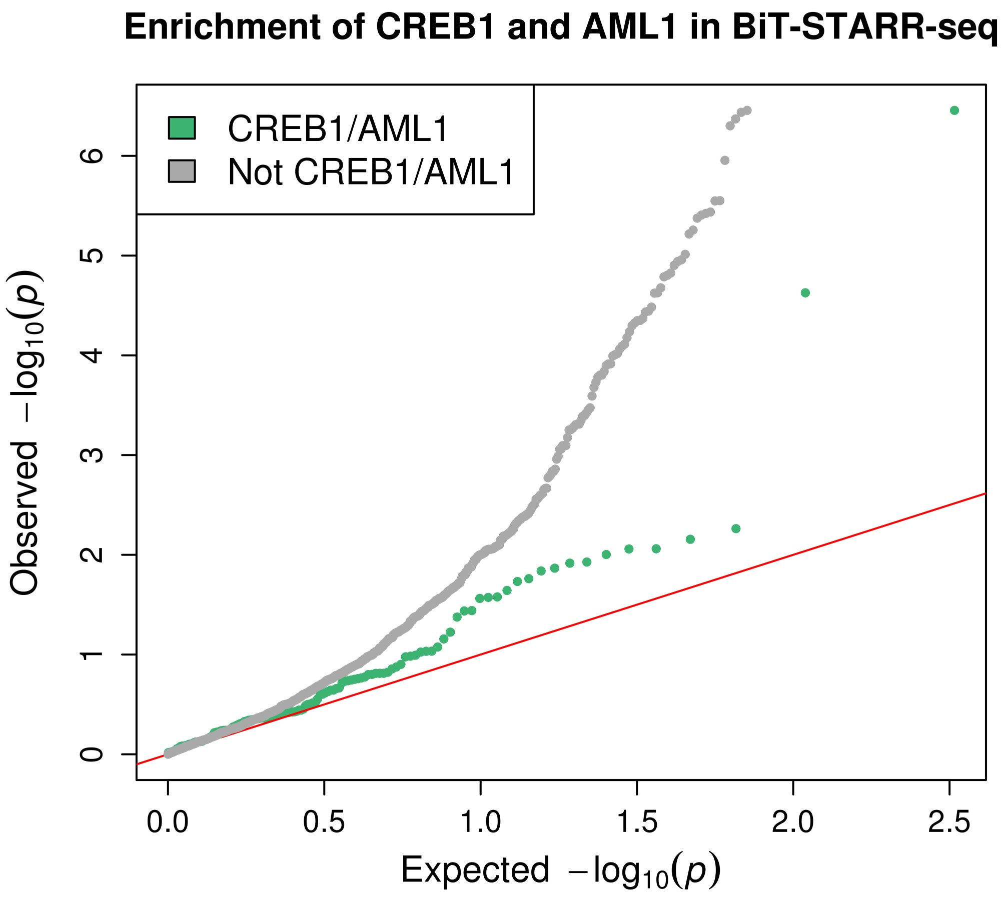

Figure 10: **Depletion of ASE with TFs that repress NFKB1 binding**. QQplot depicting the ASE p-value distribution from QuASAR-MPRA for SNPs with CREB1 or AML1 binding (green) or not with CREB1 or AML1 binding (grey)

References

Benjamini Y Hochberg Y. 1995. Controlling the false discovery rate: a practical and powerful approach to multiple testing. *Journal of the royal statistical society. Series B (* **57**: 289–300.

Buenrostro J, Giresi P, Zaba L. 2013. Transposition of native chromatin for fast and sensitive epigenomic profiling of open chromatin, DNA-binding proteins and nucleosome position. *Nature methods* **10**: 1213–1218.

Huerfano S, Ryabchenko B, Forstová J. 2013. Nucleofection of expression vectors induces a robust interferon response and inhibition of cell proliferation. *DNA and cell biology* **32**: 467–79.

Innocenti F, Cooper G, Stanaway I. 2011. Identification, replication, and functional fine-mapping of expression quantitative trait loci in primary human liver tissue. *PLoS Genetics* **7**: 1–16.

Kalita CA, Moyerbrailean GA, Brown C, Wen X, Luca F, Pique-Regi R. 2017. QuASAR-MPRA: Accurate allele-specific analysis for massively parallel reporter assays. *Bioinformatics* p. btx598.

Kim D, Langmead B, Salzberg SL. 2015. HISAT: a fast spliced aligner with low memory requirements. *Nature Methods* **12**: 357–360.

Levo M, Zalckvar E, Sharon E, Dantas Machado AC, Kalma Y, Lotam-Pompan M, Weinberger A, Yakhini Z, Rohs R, Segal E. 2015. Unraveling determinants of transcription factor binding outside the core binding site. *Genome research* **25**: 1018–29.

Li H. 2013. Aligning sequence reads, clone sequences and assembly contigs with BWA-MEM. *ArXiv* **1303**.

Love MI, Huber W, Anders S. 2014. Moderated estimation of fold change and dispersion for RNA-seq data with DESeq2. *Genome Biology* **15**: 550.

MacArthur J, Bowler E, Cerezo M, Gil L, Hall P, Hastings E, Junkins H, McMahon A, Milano A, Morales J, et al.. 2017. The new NHGRI-EBI Catalog of published genome-wide association studies (GWAS Catalog). *Nucleic acids research* **45**: D896–D901.

Moyerbrailean G, Richards A, Kurtz D, Kalita CA, Davis G, Harvey C, Alazizi A, Watza D, Sorokin Y, Hauff N, et al.. 2016a. High-throughput allele-specific expression across 250 environmental conditions. *Genome Research* **26**.

Moyerbrailean GA, Davis GO, Harvey CT, Watza D, Wen X, Pique-Regi R, Luca F. 2015. A high-throughput RNA-seq approach to profile transcriptional responses. *Scientific reports* **5**: 14976.

Moyerbrailean GA, Kalita CA, Harvey CT, Wen X, Luca F, Pique-Regi R. 2016b. Which Genetics Variants in DNase-Seq Footprints Are More Likely to Alter Binding? *PLoS Genetics* **12**: e1005875.

Muerdter F, Boryń ŁM, Woodfin AR, Neumayr C, Rath M, Zabidi MA, Pagani M, Haberle V, Kazmar T, Catarino RR, et al.. 2017. Resolving systematic errors in widely used enhancer activity assays in human cells. *Nature Methods* **15**: 141–149.

Pickrell J. 2014. Joint analysis of functional genomic data and genome-wide association studies of 18 human traits. *The American Journal of Human Genetics* **94**: 559–573.

Pique-Regi R, Degner J, Pai A, Gaffney D. 2011. Accurate inference of transcription factor binding from DNA sequence and chromatin accessibility data. *Genome research* **21**: 447–455.

Smith T, Heger A, Sudbery I. 2017. UMI-tools: modeling sequencing errors in Unique Molecular Identifiers to improve quantification accuracy. *Genome research* **27**: 491–499.

STOUFFER SA, SUCHMAN EA, DEVINNEY LC, STAR SA, WILLIAMS RMJ. 1949. The American soldier: Adjustment during army life. *Princeton University Press* **265**: 173–175.

Wen X, Luca F, Pique-Regi R. 2015. Cross-population Joint Analysis of eQTLs: Fine Mapping and Functional Annotation. *PLoS Genetics* **11**: 1–29.

Yang J, Weedon MN, Purcell S, Lettre G, Estrada K, Willer CJ, Smith AV, Ingelsson E, O 'connell JR, Mangino M, et al.. 2011. Genomic inflation factors under polygenic inheritance. *European Journal of Human Genetics* **16**.
