# Supplementary figures and images for: High-throughput characterization of genetic effects on DNA–protein binding and gene transcription
Source: Genome Res. 2018 Nov;28(11):1701–8. doi: 10.1101/gr.237354.118 (PMC6211638; doi:10.1101/gr.237354.118)

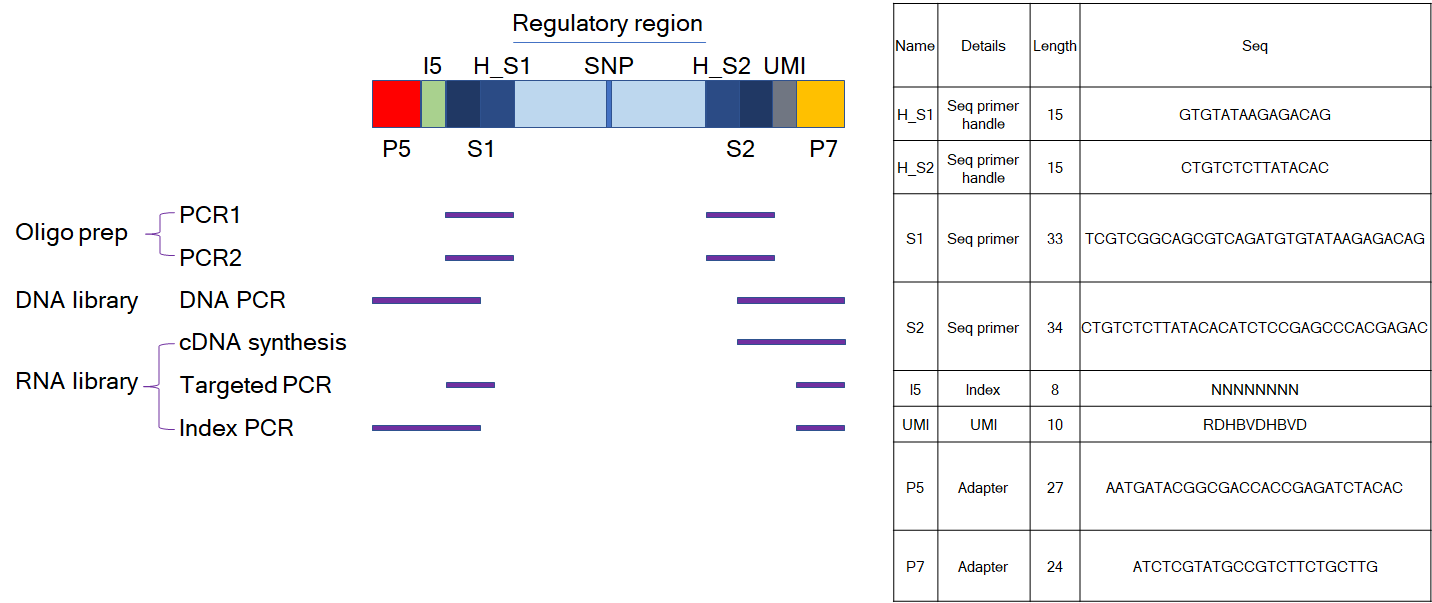

Supplement: Supplemental Material [file supp_gr.237354.118_Supplemental_Fig_S1.png]

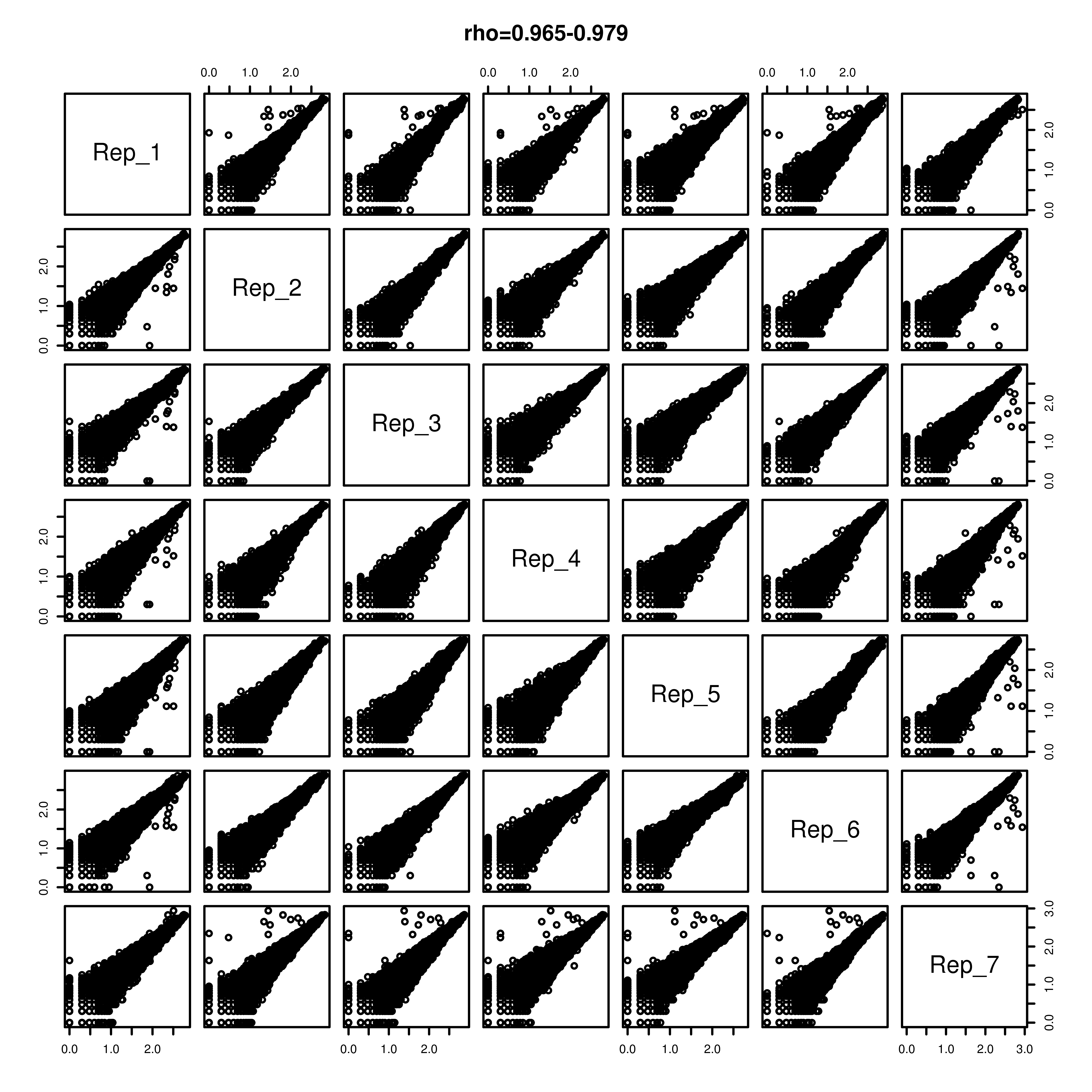

Supplement: Supplemental Material [file supp_gr.237354.118_Supplemental_Fig_S2.png]

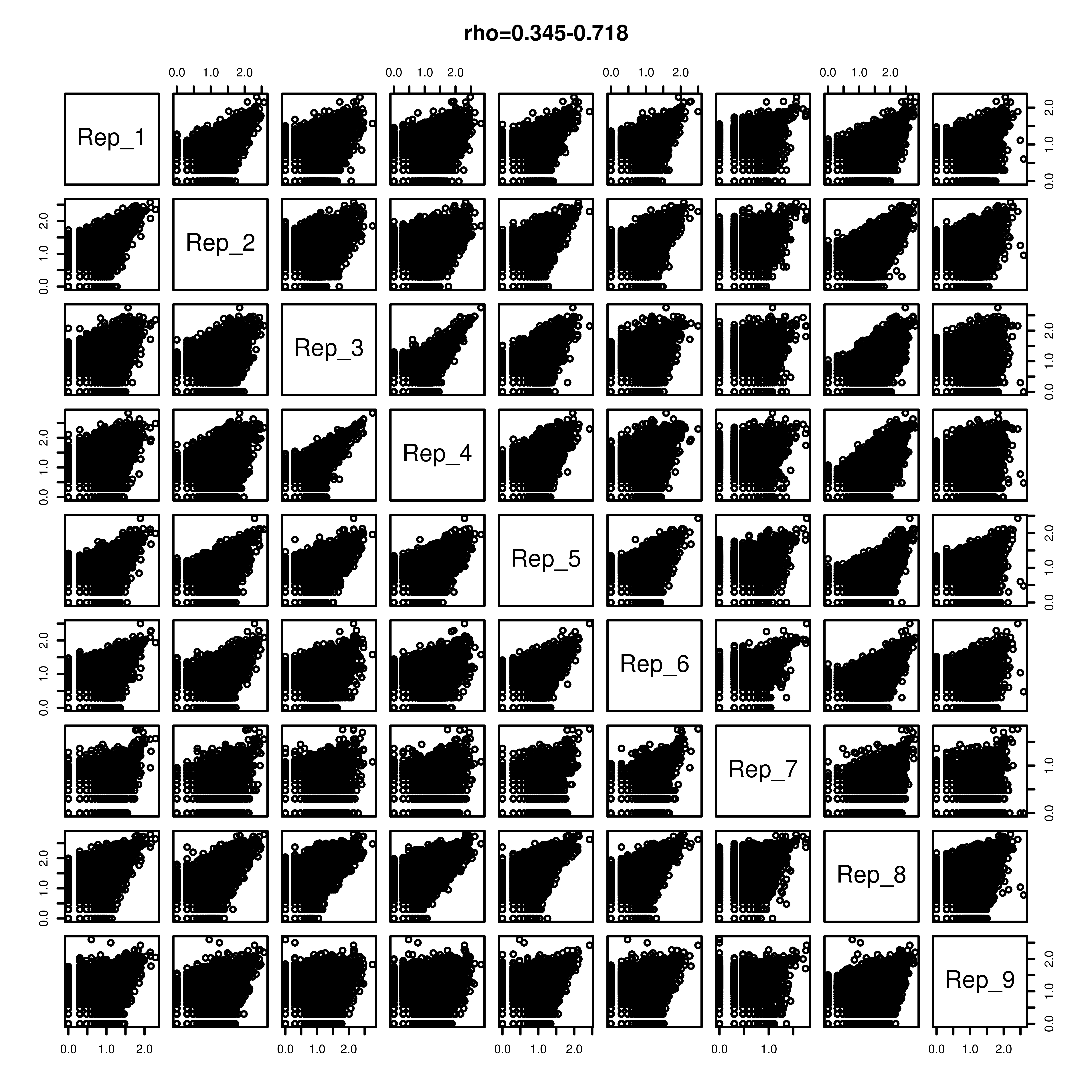

Supplement: Supplemental Material [file supp_gr.237354.118_Supplemental_Fig_S3.png]

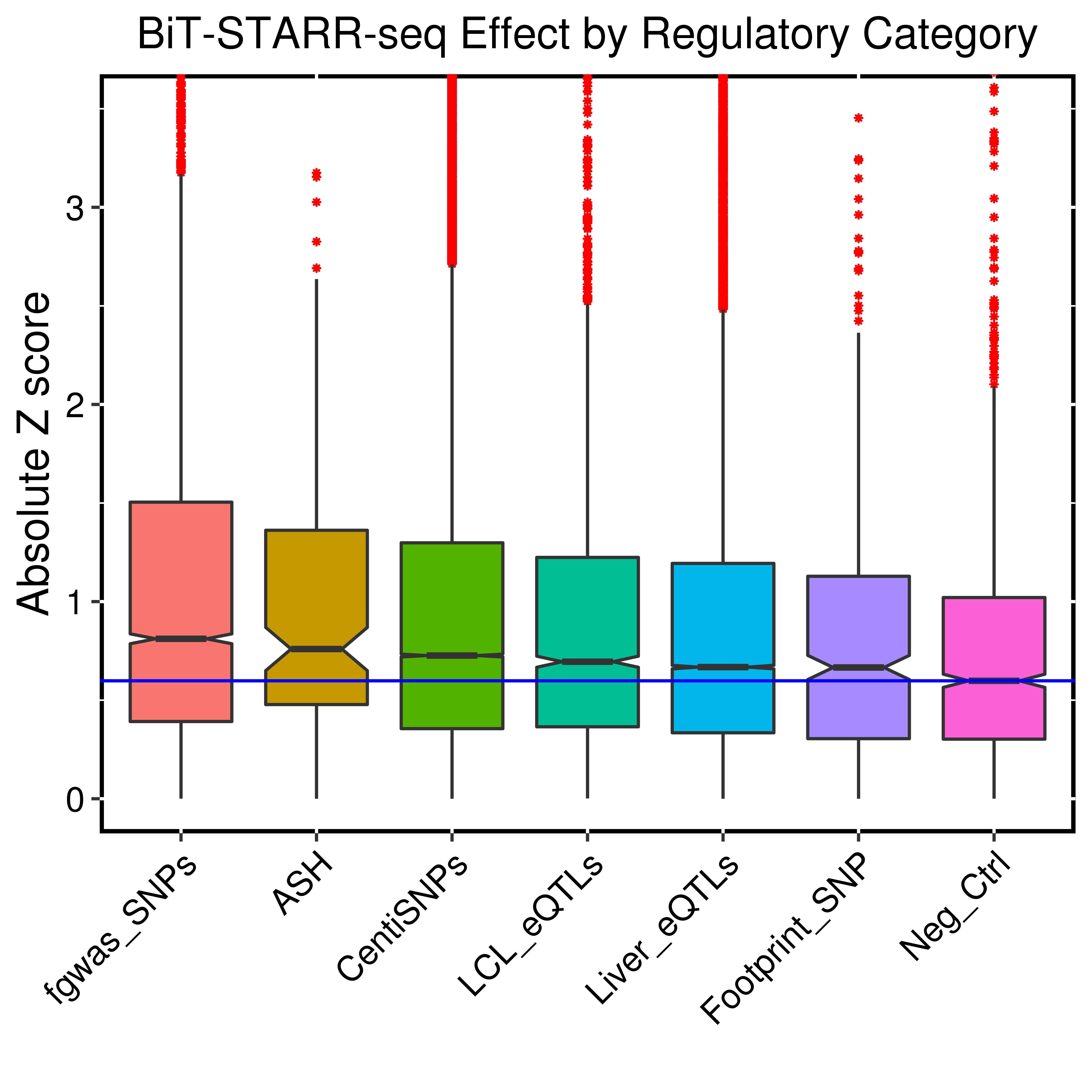

Supplement: Supplemental Material [file supp_gr.237354.118_Supplemental_Fig_S4.png]

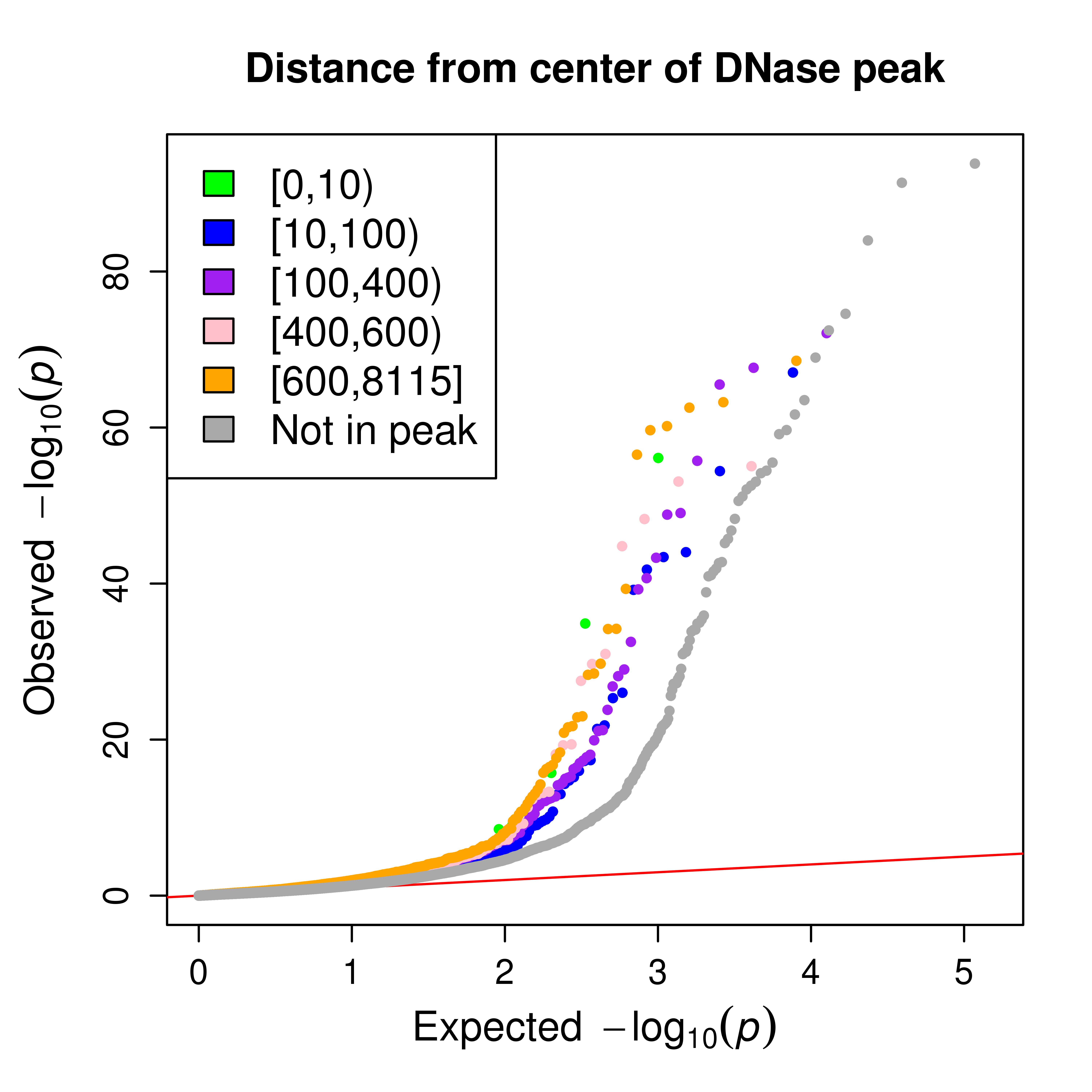

Supplement: Supplemental Material [file supp_gr.237354.118_Supplemental_Fig_S5.png]

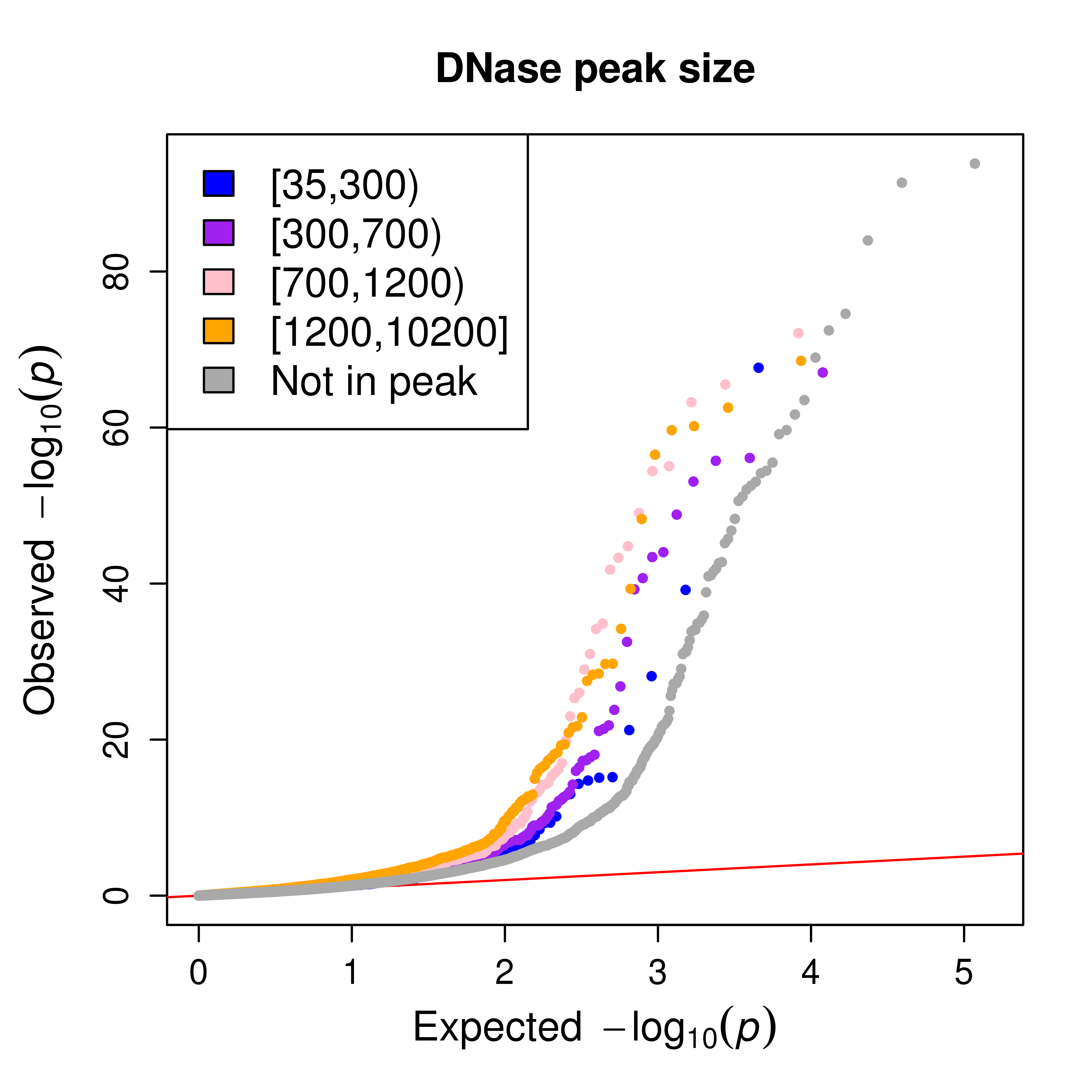

Supplement: Supplemental Material [file supp_gr.237354.118_Supplemental_Fig_S6.png]

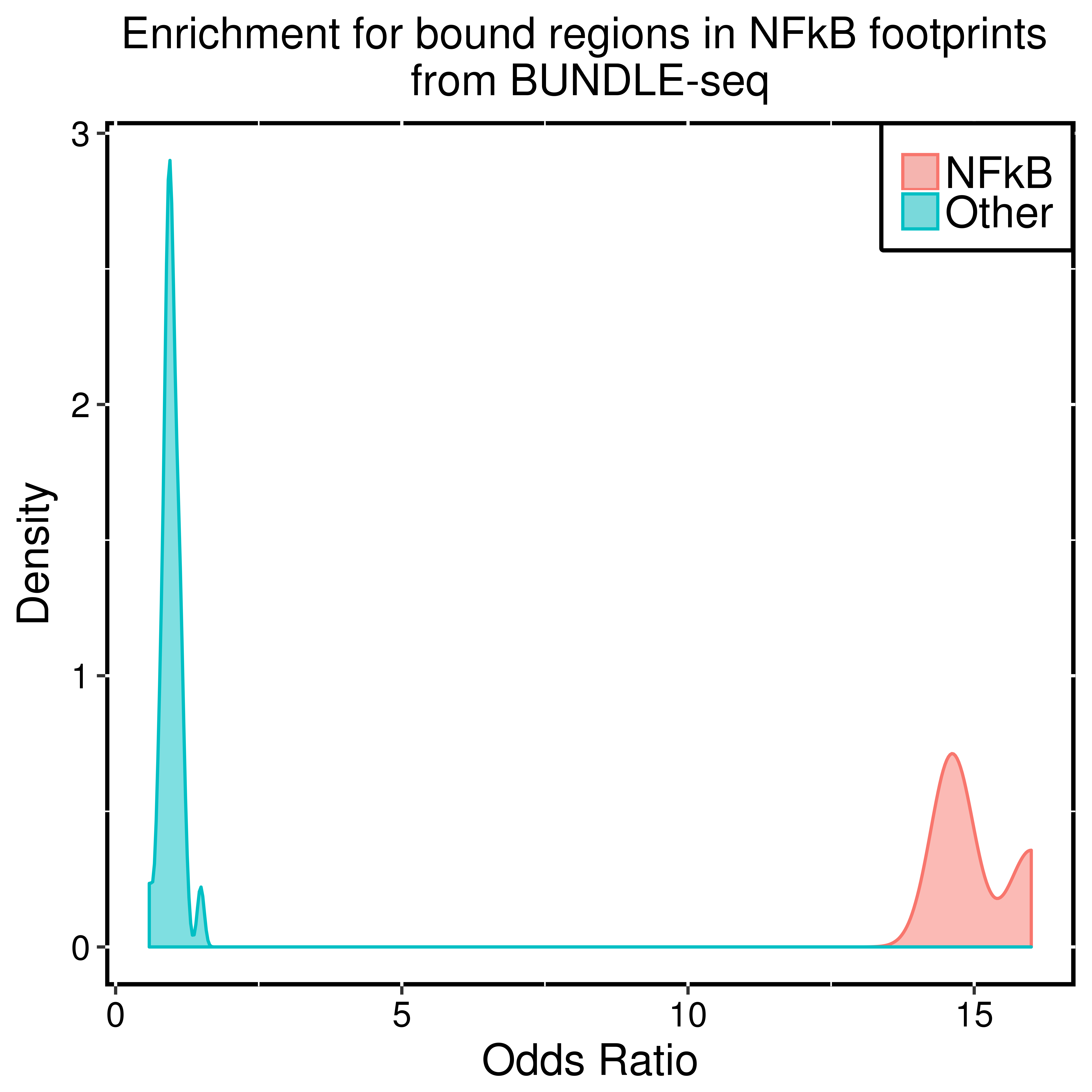

Supplement: Supplemental Material [file supp_gr.237354.118_Supplemental_Fig_S7.png]

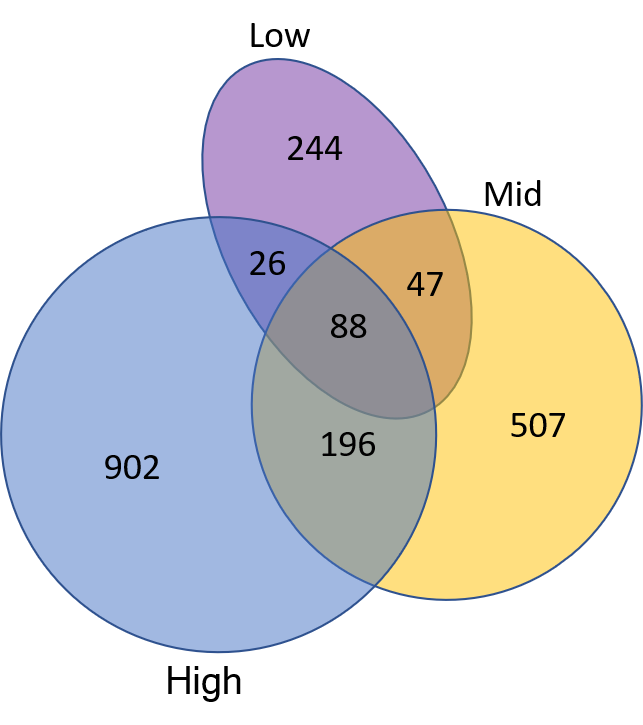

Supplement: Supplemental Material [file supp_gr.237354.118_Supplemental_Fig_S8.png]

A

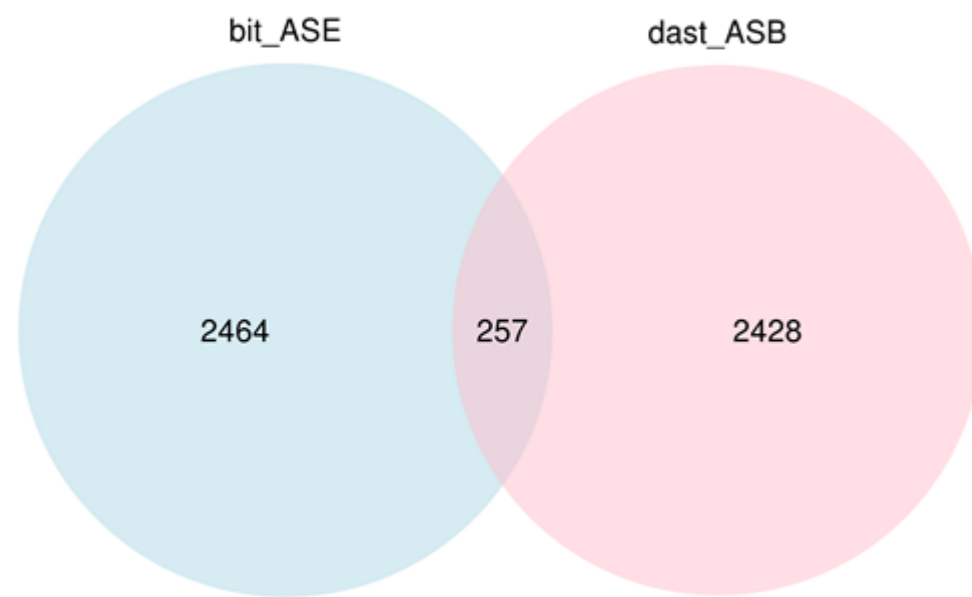

B

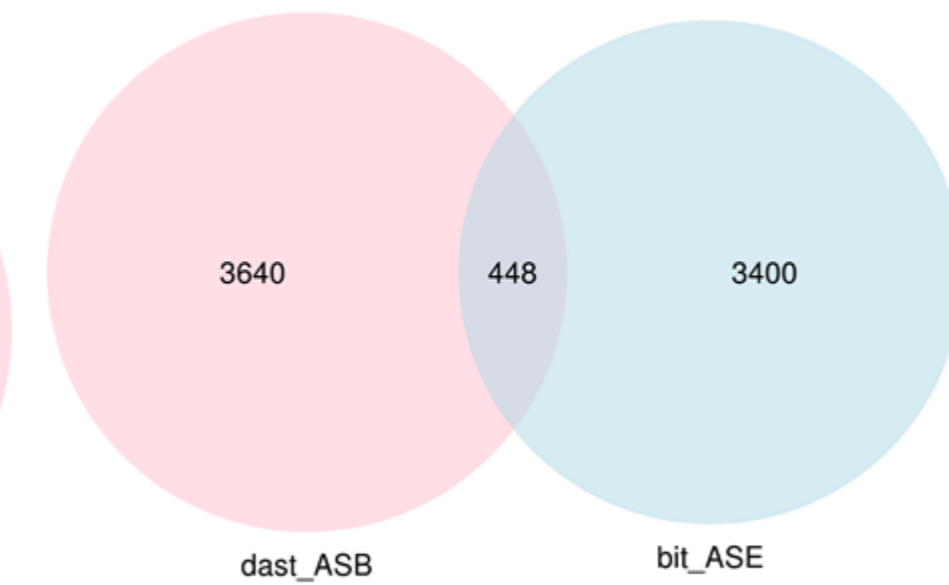

C

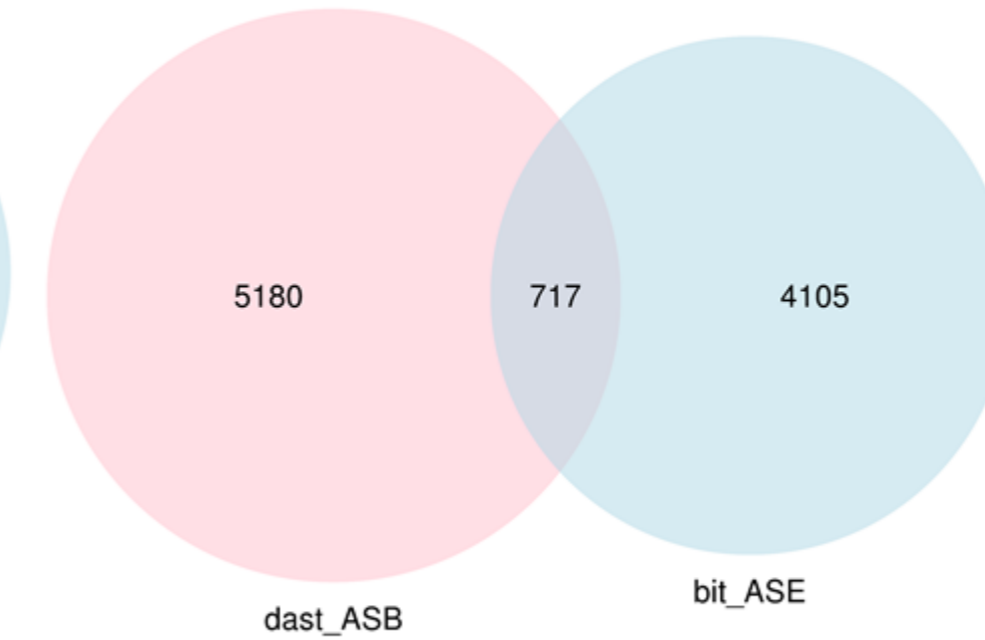

D

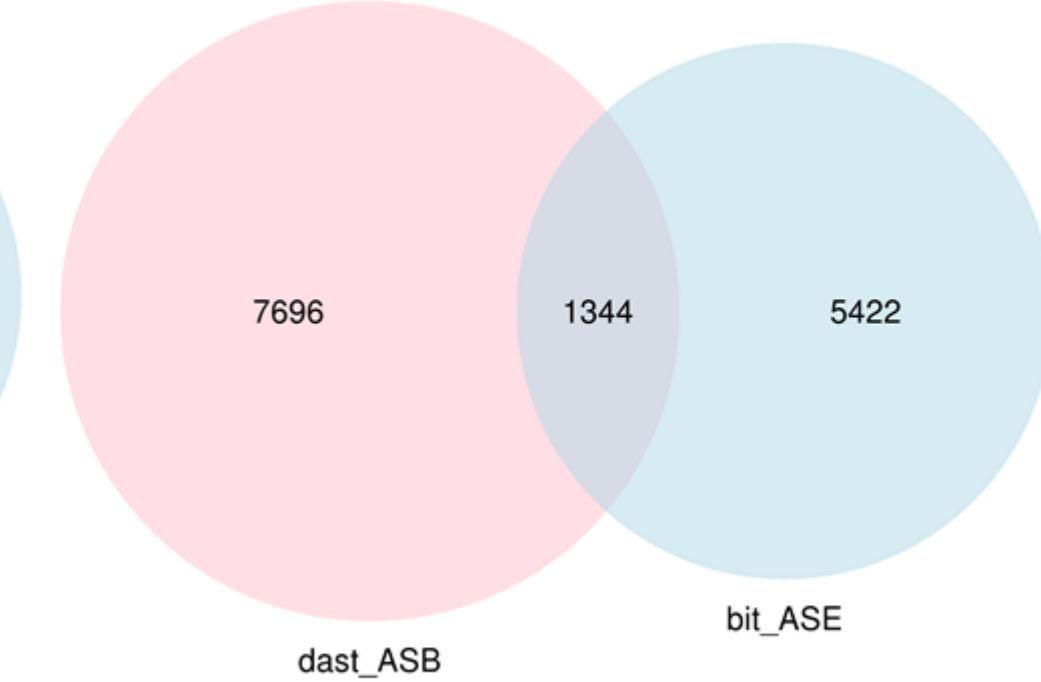

Supplement: Supplemental Material [file supp_gr.237354.118_Supplemental_Fig_S9.pdf]

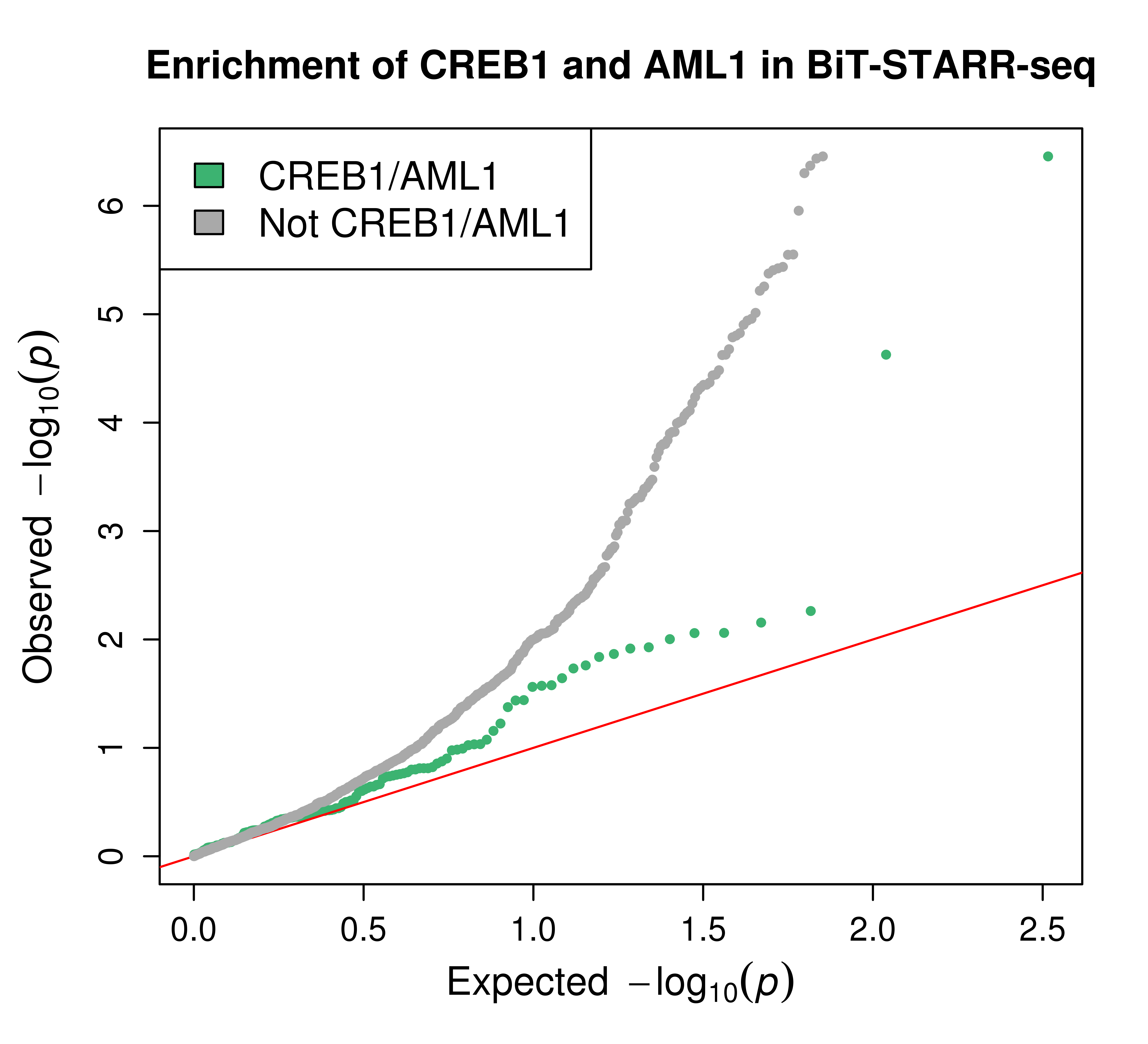

Supplement: Supplemental Material [file supp_gr.237354.118_Supplemental_Fig_S10.png]
