# Supplementary material for: High-throughput characterization of genetic effects on DNA–protein binding and gene transcription
Source: Genome Res. 2018 Nov;28(11):1701–8. doi: 10.1101/gr.237354.118 (PMC6211638; doi:10.1101/gr.237354.118)
Supplement: Supplemental Material [file supp_gr.237354.118_Supplemental_Methods_S2.pdf]

BiT-STARR-seq Protocol

Table 1: **Primers used in BiT-STARR-seq**

Primer	Sequence
STARR_F_SH	CCGAGCCCACGAGACCTAGAGTCGGGGCGGCCG
STARR_R_SH	TGACGCTGCCGACGAAATTATTACACGGCGATC
F_transposase	TCGTCGGCAGCGTCAGATGTGTATAAGAGACAG
R_transposase	GTCTCGTGGGCTCGGAGATGTGTATAAGAGACAG
F_trans_short	TCGTCGGCAGCGTCAGAT
I2.1	CAAGCAGAAGACGGCATACGA
Nextera_i7_10N	CAAGCAGAAGACGGCATACGAGAT**RDHBVDHBVD**GTCTCGTGGGCTCGG

.

Part 1

PCR to double strand oligo and fill out sequencing primer

1. Resuspend oligos to 10ng/μl WS
2. PCR1 prep samples

Component	20 μl Reaction	Final Concentration
Nuclease-free water	6.4 μl	
10 μM F_transposase	1 μl	0.5 μM
10 μM R_transposase	1 μl	0.5 μM
Template DNA	1 μl oligo WS	10ng
DMSO	0.6 μl	3%
2X Phusion Master Mix	10 μl	1X

Gently mix the reaction. Collect all liquid to the bottom of the tube by a quick spin if necessary. cycling conditions: 98°C for 30s, followed by 4 cycles of 98°C for 10s, 50°C for 30s, 72°C for 60s, followed by 6 cycles of 98°C for 10s, 65°C for 30s, 72°C for 60s, followed by 72°C for 5 min

3. Nucleospin gel and PCR cleanup
    a. Run 2% gel 100V 50min

PCR to amplify oligos

4. PCR2

Component	Volume	Final concentration
Nuclease-free water	6.4 μl	
10 μM F_transposase	1 μl	0.5 μM
10 μM R_transposase	1 μl	0.5 μM
Template DNA	dilute PCR1 1:5, then use 1 μl	
DMSO	0.6 μl	3%
2X Phusion Master Mix	10 μl	1X

Gently mix the reaction. Collect all liquid to the bottom of the tube by a quick spin if necessary. cycling conditions: 98°C for 30s, followed by 15 cycles of 98°C for 10s, 50°C for 30s, 72°C for 60s, followed by 72°C for 5 min

5. Nucleospin gel and PCR cleanup
    a. Run 2% gel 100V 50min
    b. Nanodrop

Prepare plasmid for cloning

6. Linearize pGL4.23

Component	Volume	Final Concentration
5 μM STARR_F_SH	1.5 μl	0.20.3 μM
5 μM STARR_F_SH	1.5 μl	0.20.3 μM
1 ng/μl pGL4.23	1 μl	
Nuclease-free water	8.5 μl	
CloneAmp HiFi PCR Premix	12.5 μl	1X

Gently mix the reaction. Collect all liquid to the bottom of the tube by a quick spin if necessary. cycling conditions: 35 cycles of 98°C for 10s, 60°C for 15s, and 72°C for 5s.

7. Nucleospin gel and PCR cleanup
    a. Run 1% gel 100V 60min

Cloning

8. Clone libraries with putative enhancer sequences into linearized pGL4.23 plasmid
   a. Set up cloning reactions: (36 rxns per set + pos and neg ctrls)

Component	Cloning Rxn	Negative control	Positive control
PCR2 oligos	25ng	NA	2 μl of 2kb control insert
Linear Vector	100ng	1 μl	1 μl of pUC19 control vector
Premix	2 μl	2 μl	2 μl
Water	to 10 μl	to 10 μl	to 10 μl

   b. Incubate for 15 min at 50°C then place on ice.

   c. Cleanup using Pureyield Miniprep columns
      i. Add cloning rxns (36) to spin column
      ii. Spin at full speed (20000 rcf) for 15 sec
      iii. Add 400 μl wash buffer, and spin at full speed for 15 sec
      iv. Empty column and spin additional 30sec
      v. Add 30 μl elution buffer, sit 1 min, spin at full speed 1 min

Transformation

9. Transformation using XL-10 gold competent cells (1x).
   a. Prior to start: Make LB with amp plates
   b. 100 μl cells thawed on ice. Add 4 μl B-mercap. (in 14ml tube)
   c. Ice 10min, occasional mixing.
   d. Add 2 μl cloning rxn
   e. Ice 30min. Take out SOC to thaw.
   f. Heat shock 30sec at 42°C (water bath)
   g. Ice 2min
   h. Add 900 μl SOC
   i. Shaker 1 hr (37°C)
   j. Plate 1 μl (+99 μl SOC)
   k. Incubate overnight, count colonies *hoping to get 200colonies (or more)
   l. Calculate complexity (example below)

Colonies*dilution*plated V
200*1000 μl*1 μl = 200k
105k constructs*(want to see 5x)*(2 directions)=1050k
1050000/200000=5.25 transformations *(2 for safety) = 10.5 transformations

10. Repeat transformation doing full replicates.
   a. Combine transformations. Plate 1 μl on LB plate + amp, and incubate overnight. Place remainder in LB broth + amp and grow overnight at 37°C in shaker. 200ml per flask (want 4x).
   b. Check that plate has good colony growth. (optional: keep for miniprep and sanger seq confirmation)

11. Maxiprep (4x) using Qiagen Endo-free maxiprep kit (no changes made to standard protocol)
   a. Nanodrop. Aliquot.

Part 2

1. Nucleofection LCLs

a. Prior to Start:

i. media bottle in water bath

ii. Prepare DNA (want [3 μg] * number of cuvettes (3 per replicate)) and aliquot into microcentrifuge tubes (1 per replicate)

iii. Prepare required amount of solution+supplement (ratio 1:4.5). 82 μl SE solution:18 μl supplement 1 per cuvette

b. Count cells

c. Spin down required amount (7.5e6 per cuvette) at 90G for 10 min

d. Resuspend cells in solution +supplement (100 μl per cuvette)

e. Add cells to DNA tubes

f. Dispense into cuvette (max volume 110 μl)- make sure they are all the way in the bottom groove and tap lightly on the hood

g. Electroporate with correct program (DS-150 and SE solution)

h. Let sit at room temperature for 10 min

i. Add media to cuvette pipetting into the side of the well gently (450 μl)

j. Incubate at 37°C for 30min

k. Mix cells once gently, then seed into T75 flask (1 per replicate, 27ml total volume)

l. Incubate 24 hrs

m. Lysis

i. Pellet by centrifugation (e.g., 400 x g for 8 minutes at 4°C).

ii. Wash the pellets by re-suspending in ice cold phosphate-buffered saline (PBS), 5ml. Pellet cells by centrifugation again. Gently aspirate the supernatant from each tube.

iii. Make LysisBinding Buffer (RT): Add 10 μl b-ME per 1 ml Buffer RLT Plus (want 1.8ml per replicate).

iv. Add the appropriate volume of LysisBinding Buffer (RT) to each replicate.

v. Triturate the solution through a pipette tip to obtain complete lysis. The release of DNA during lysis results in a viscous solution which confirms complete lysis.

vi. Freeze at -80°C to aid in the lysis step (at least 1 day).

DNA Library Prep

1. Prepare the PCR reaction mix to add the index adaptors to the DNA.

Component	Volume
Nuclease-free water	10 μl
N5xx Index Adaptor	2.5 μl
15 μM Nextera_i7_10N	2.5 μl
DNA (Maxiprepped plasmid pool)	50ng
NEB Next 2x Master Mix	25 μl

Gently mix the reaction. Collect all liquid to the bottom of the tube by a quick spin if necessary. cycling conditions: 72°C for 5 min followed by 98°C for 30s, followed by 5 cycles of 98°C for 10s, 63°C for 30s, 72°C for 60s.Hold at 4°C.

Preform side reaction to determine number of cycles to add:

Component	Volume
Nuclease-free water	3 μl
N5xx Index Adaptor	0.5 μl
15 μM Nextera_i7_10N	0.5 μl
5 cycle-PCR amplified DNA	5 μl
10X SYBR Green I	1 μl
NEB Next 2x Master Mix	5 μl

Gently mix the reaction. Collect all liquid to the bottom of the tube by a quick spin if necessary. cycling conditions: 20 cycles of 98°C for 10s, 63°C for 30s, 72°C for 60s. Calculate 25% max threshold and finish PCR cycles.

2. Nucleospin gel and PCR cleanup
   a. Run 2% gel 100V 50min

3. QC:
   a. Run on bioanalyzer to check peak is correct (about 350bp)
   b. Run Kappa quant qPCR to get library concentration for loading.

RNA Library Prep

1. Total RNA extraction

a. Pipet the lysate (600 μl) directly into a QIAshredder spin column placed in a 2 ml collection tube, and centrifuge for 2 min at full speed (17900 rcf).

b. Transfer homogenized lysate to a gDNA Eliminator spin column, and centrifuge 30sec at full speed. Discard column and SAVE flow through.

c. Add 600 μl (1 volume) of 70% ethanol to the flow-through, and mix well by pipetting. Do not centrifuge.

d. Transfer up to 700 μl of the sample, including any precipitate, to an RNeasy spin column placed in a 2 ml collection tube (supplied). Close the lid, and centrifuge for 15 s full speed. Discard the flow-through.

e. Add 700 μl Buffer RW1 to the RNeasy Mini spin column (in a 2 ml collection tube). Close the lid, and centrifuge for 15 s at full speed. Discard the flow-through

f. Add 500 μl Buffer RPE to the RNeasy spin column. Close the lid, and centrifuge for 15 s at full speed. Discard the flow-through.

g. Add 500 μl Buffer RPE to the RNeasy spin column. Close the lid gently, and centrifuge for 2 min at full speed.

h. Place the RNeasy spin column in a new 2 ml collection tube (supplied). Centrifuge at full speed for 1 min to further dry the membrane.

i. Place the RNeasy spin column in a new 1.5 ml collection tube (supplied). Add 52 μl RNase-free water directly to the spin column membrane. Wait 1 min. Close the lid, and centrifuge for 1 min at full speed to elute the RNA.

j. Add 52 μl RNase-free water directly to the spin column membrane. Wait 1 min. Close the lid, and centrifuge for 1 min at full speed to elute the RNA.

2. Total RNA to mRNA

a. Resuspend Dynabeads Oligo(dT)25 thoroughly before use.

b. Transfer 200 μl of Dynabeads from the stock tube to each well on an RNase-free 96-well plate and place the plate on the magnet.

c. After 30 seconds (or when the suspension is clear), remove the supernatant.

d. Remove the plate from the magnet and wash the beads by re-suspending in 200 μl of fresh LysisBinding Buffer.

e. Adjust the volume of up to 75 μg total RNA to 100 μl with 10 mM Tris-HCl, pH 7.5 (should already be at 100 μl)

f. Heat to 65°C for 2 minutes to disrupt secondary structures. Place on ice.

g. Remove the Lysis/Binding Buffer from the prewashed Dynabeads Oligo(dT)25 by placing on the magnet for 30 seconds, or until the suspension is clear and discard the supernatant.

h. Remove the plate from the magnet and add 100 μl total RNA plus 100 μl LysisBinding Buffer.

i. Pipet to resuspend the beads completely in the sample lysate.

j. Seal the plate and incubate with continuous mixing in the heat block for 5 min, at 25°C and 1000 rpm, to allow the polyA tail of the mRNA to hybridize to the oligo (dT)25 on the beads.

k. Place the plate on the magnet for 2 minutes and remove the supernatant. If the solution is noticeably viscous, increase the time to approximately 10 minutes.

l. Set the heat block to 80°C for later step.

m. Remove the beads from the magnet and wash the beads/ mRNA complex two times

n. Wash the beads/mRNA complex with 200 μl Washing Buffer B at RT, place on magnet 5 minutes. Be sure to RINSE the SIDES OF THE WELLS with Washing Buffer B.

o. Collect the mRNA elution from the beads by adding 10 μl of 10mM Tris-HCl pH 7.5 (Elution Buffer), mixing well, and incubate at 80°C for 2 minutes.

p. Immediately place the plate on the magnet, about 1 min wait and transfer the supernatant (9 μl) containing the mRNA to a new RNAse-free plate and place on ice. Note: Take 1 μ l from each well into a plate for quantification.

cDNA synthesis

3. cDNA synthesis using Superscript III kit standard protocol.

4. Purify the cDNA using SPRI Beads

a. Swirl SPRI beads to resuspend.

b. Pool cDNA reactions for the same replicate to get 60ul final volume.

c. Add 54 μl (0.9X) of resuspended SPRI beads to the pooled cDNA reaction. Mix well on a vortex mixer or by pipetting up and down at least 10 times.

d. Cover the plate with an aluminum PCR seal and incubate for 5 minutes at room temperature.

e. Place the plate on an appropriate magnetic stand to separate beads from the supernatant. After the solution is clear (about 2 minutes), carefully remove and discard the supernatant. Be careful not to disturb the beads that contain DNA targets.

f. Add 200 μl of freshly prepared 80% ethanol to each well of the plate while in the magnetic stand. Incubate at room temperature for 30 seconds, and then carefully remove and discard the supernatant.

g. Repeat Step 5 once for a total of 2 washing steps.

h. Air-dry the beads for 2 minutes, while the plate is on the magnetic stand uncovered.

i. Elute the DNA target from the beads into 21 μl water. Mix well on a vortex mixer or by pipetting up and down. Cover the plate with an aluminum PCR seal and quickly spin the plate in a microcentrifuge and place it on the magnetic stand until the solution is clear.

j. Transfer 20 μl of the supernatant to a clean PCR plate.

PCR to target oligo in cDNA and amplify

5. Targeted PCR

Component	Volume
10 μM F_trans_short	1.7 μl
10 μM I_2.1	1.7 μl
Pure cDNA	20 μl
DMSO	1.5 μl
2X Phusion Master Mix	25 μl

Include DNA positive control (3 μl DNA library and 17 μl nuclease free water) to use as a marker for size selection on the gel. Gently mix the reaction. Collect all liquid to the bottom of the tube by a quick spin if necessary.
cycling conditions: 98°C for 30s, followed by 15 cycles of 98°C for 10s, 72°C for 15s, followed by 72°C for 5 min

6. Nucleospin gel and PCR cleanup
   a. Run 2% gel 140V 2 hrs

PCR to add multiplexing index and amplify

7. Index PCR

Component	Volume
N5xx Index Adaptor	1.7 μl
15 μM I_2.1	1.7 μl
Pure targeted PCR	20 μl
DMSO	1.5 μl
2X Phusion Master Mix	25 μl

Gently mix the reaction. Collect all liquid to the bottom of the tube by a quick spin if necessary. cycling conditions: 98°C for 30s, followed by 5 cycles of 98°C for 10s, 72°C for 15s, followed by 72°C for 2 min

Preform side reaction to determine number of cycles to add:

Component	Volume
Nuclease-free water	3 μl
N5xx Index Adaptor	0.5 μl
15 μM I_2.1	0.5 μl
5 cycle-PCR amplified DNA	5 μl
10X SYBR Green I	1 μl
2X Phusion Master Mix	5 μl

Gently mix the reaction. Collect all liquid to the bottom of the tube by a quick spin if necessary. cycling conditions: 30 cycles of 98°C for 10s, 72°C for 15s.
Calculate 25% max threshold and finish PCR cycles. X cycles of 98°C for 10s, 72°C for 15s, followed by 72°C for 5 min.

8. Nucleospin gel and PCR cleanup
   a. Run 2% gel 140V 2 hrs

9. QC:
   a. Run on bioanalyzer to check peak is correct (about 350bp)
   b. Run Kappa quant qPCR to get library concentration for loading.
